# Effect of smoking on prostate cancer: Results from the National Health and Nutrition Examination Survey 2003–2018 and Mendelian randomization analyses

**DOI:** 10.18332/tid/189199

**Published:** 2024-06-04

**Authors:** Hairong He, Liang Liang, Tao Tian, Xiaoyu Zhang, Jun Lyu

**Affiliations:** Department of Hepatobiliary Surgery, The First Affiliated Hospital of Xi'an Jiaotong University, Xi'an, China; Department of Urology, The First Affiliated Hospital of Xi'an Jiaotong University, Xi'an, China; Department of Medical Oncology, The First Affiliated Hospital of Xi'an Jiaotong University, Xi'an, China; Department of Clinical Research, The First Affiliated Hospital of Jinan University, Guangzhou, China

**Keywords:** smoking, prostate cancer, NHANES, Mendelian randomization

## Abstract

**INTRODUCTION:**

The controversial relationship between smoking and prostate cancer (PCa) risk prompted us to conduct a cross-sectional study using the National Health and Nutrition Examination Survey (NHANES) database and apply Mendelian randomization (MR) analyses in order to clarify the possible causal effect of smoking on PCa risk.

**METHODS:**

Using univariate and multivariate logistic regression methods, a secondary analysis of the pooled 2003–2018 NHANES dataset was performed to explore the association between smoking and PCa risk. Propensity-score matching was used to reduce selection bias. Then, we conducted subsequent MR analysis study to investigate the potential causal effect of smoking on PCa risk, with genetic variants of four exposure factors including the lifetime smoking index, light smoking, smoking initiation, and the amount of smoking per day obtained from genome-wide association studies, and PCa summary statistics obtained from three database populations. Inverse-variance weighting was the primary analytical method, and weighted median and MR-Egger regression were used for sensitivity analyses. The MR results for the three PCa databases were combined using meta-analysis.

**RESULTS:**

The study included 16073 NHANES subjects, comprising 554 with PCa and 15519 without PCa. Logistic regression before and after matching did not reveal any significant association. Meta-analysis of the MR results also did not support an association of PCa risk with lifetime smoking index (OR=0.95; 95% CI: 0.83–1.09), light smoking (OR=1.00; 95% CI: 0.95–1.06), smoking initiation (OR=0.99, 95% CI=0.99–1.00), or the amount of smoking per day (OR=1.00; 95% CI: 0.99–1.00) and PCa risk.

**CONCLUSIONS:**

There was no evidence for an association between smoking and the risk of PCa. Further studies are needed to determine if there are any associations of other forms of smoking with the risk of PCa at different stages.

## INTRODUCTION

The association of smoking with prostate cancer (PCa) remains disputed due to different conclusions coming from previous studies. Most epidemiology studies have found no association^1^, but there have been several reports of a positive association^2^, with some studies even finding that smoking may exert a protective effect against the PCa risk^3,4^. These contradictory findings indicate that the effect of smoking on PCa incidence needs to be investigated further while taking into account that the contradictory results may stem largely from differences in the definition of smoking, race of participants, and research period^3^.

The National Health and Nutrition Examination Survey (NHANES) is a nationally representative survey of American civilians that provides comprehensive data on various aspects of health and nutrition^5^. The survey is unique in combining interviews and physical examinations. NHANES, therefore, provides high-quality and nationally representative data that can be used to determine the prevalence and risk factors for diseases. Although NHANES has a retrospective design and bias is inevitable, its comprehensive nature means that possible confounding factors can be controlled^6^. Cigarette smoking is the predominant mode of tobacco consumption, and the present cross-sectional study is the first to investigate the association of cigarette smoking with the risk of PCa using NHANES data.

Mendelian randomization (MR) is an epidemiological method that utilizes genetic variants as instrumental variables for quantifying exposure and can be used to estimate the potential causal role of exposure in disease development. The MR design mitigates confounding since the genetic variants are assorted randomly during gamete formation and are mostly independent of environmental and lifestyle factors^7^. In this study, we perform a two-sample MR analysis intending to clarify whether there are potential causal effects of cigarette smoking on PCa risk.

## METHODS

### Study design

Firstly, a secondary dataset analysis of pooled 2003–2018 NHANES data was conducted to explore whether smoking is associated with the risk of PCa. Subsequently, we conducted Mendelian randomization analysis based on publicly available genome-wide association study (GWAS) data to clarify the possible causal effect of smoking on PCa risk at the genetic level.

### Cross-sectional study using the NHANES database

*Study population in NHANES*

NHANES has a 2-year-cycle cross-sectional design. The population included in this study comprised male responders who either had or had not received a PCa diagnosis, as determined using the following questions: ‘Have you ever been told that you had cancer or malignancy?’, ‘First cancer – what kind was it?’, ‘Second cancer – what kind was it?’, and ‘Third cancer – what kind was it?’. Responders who answered ‘yes’ to the first question and ‘prostate’ to any of the other three questions were identified as having PCa. In contrast, other responses were classified as PCa not being present. Those refusing to answer, answering ‘don't know’, or have not responded to the first question were excluded, as were responders having more than three types of cancer.

*Study variables in NHANES*

The factor investigated in this study was the smoking status, which was categorized using the following question: ‘Have you smoked at least 100 cigarettes during your life?’^8^. Those refusing to answer, answering ‘don't know’, or having missing information were excluded. Based on previous epidemiology studies^8-10^, the influencing factors that were planned to be analyzed in the present study included age, race, education level, BMI (calculated through self-reported height and weight), hypertension status, diabetes status, and dietary intakes of energy, protein, carbohydrate, total fat, total polyunsaturated fat, cholesterol, vitamin E, vitamin A, calcium, magnesium, selenium, caffeine, and alcohol. The dietary data were based on the average total nutrient intakes on the first and second days. The definitions of all variables can be found on the NHANES website (https://www.cdc.gov/nchs/nhanes/). Those with unclear information on influencing factors were excluded. Individuals with excessive energy intake (± 3 SD) were also excluded. Since there was only one day of dietary recall for individuals in the surveys conducted before 2002 and only a relatively small amount of data was available after 2019, we only included data for 2003–2018.

### Mendelian randomization study

*Selection of instrumental variables*

Smoking behaviors were categorized as follows: 1) the lifetime smoking index, as derived from the most recent GWAS in a sample of 462690 European-descent individuals that identified 126 significant single-nucleotide polymorphisms (SNPs) related to that index^11^; 2) light smoking, defined as having smoked at least 100 cigarettes during the lifetime from the GWAS pipeline using Pheasant-derived variables from UK Biobank (GWAS ID=ukb-b-8133) (https://gwas.mrcieu.ac.uk/datasets/ukb-b-8133/); 3) smoking initiation, as derived from a GWAS of Europeandescent individuals (GWAS ID=ieu-b-4877)^12^; and 4) the amount of smoking per day, as derived from a GWAS of European-descent individuals (GWAS ID=ieu-b-25)^12^. We extracted the significant variants associated with each trait (p<5×10^–8^). In addition, only those with a long physical distance (≥10000 kb) and a low probability of linkage disequilibrium (R^2^<0.001) were retained. Supplementary file Table 1 lists the instrumental variables.

**Table 1:** Baseline characteristics of the participants from the National Health and Nutrition Examination Survey 2003–2018 before and after propensity-score matching (N=16073)

*Characteristics*	*Before propensity-score matching*				*After propensity-score matching*			
	*Non-PCa (N=15519) n (%)*	*PCa (N=554) n (%)*	*χ^2^/t*	*p*	*Non-PCa (N=554) n (%)*	*PCa (N=554) n (%)*	*χ^2^/t*	*p*
**Age** (years), mean ± SD	49.89 ± 17.69	72.62 ± 7.75	-30.13	<0.01	72.66 ± 7.7	72.62 ± 7.75	0.09	0.93
Race			84.94	<0.01			7.92	0.09
Mexican American	2304 (14.85)	23 (4.15)			21 (3.79)	23 (4.15)		
Other Hispanic	1203 (7.75)	31 (5.6)			30 (5.42)	31 (5.6)		
Non-Hispanic White	7246 (46.69)	316 (57.04)			337 (60.83)	316 (57.04)		
Non-Hispanic Black	3248 (20.93)	157 (28.34)			124 (22.38)	157 (28.34)		
Other race including multi-racial	1518 (9.78)	27 (4.87)			42 (7.58)	27 (4.87)		
Education level			13.59	<0.01			2.69	0.61
Lower than 9th grade	1520 (9.79)	63 (11.37)			62 (11.19)	63 (11.37)		
9–11th grade (includes 12th grade with no diploma)	2154 (13.88)	69 (12.45)			75 (13.54)	69 (12.45)		
High school graduate/GED or equivalent	3775 (24.33)	114 (20.58)			133 (24.01)	114 (20.58)		
Some college or AA degree	4347 (28.01)	143 (25.81)			132 (23.83)	143 (25.81)		
College graduate or higher	3723 (23.99)	165 (29.78)			152 (27.44)	165 (29.78)		
Hypertension			211.27	<0.01			2.64	0.1
Yes	5549 (35.76)	366 (66.07)			340 (61.37)	366 (66.07)		
No	9970 (64.24)	188 (33.94)			214 (38.63)	188 (33.94)		
Diabetes			32.51	<0.01			5.99	0.05
Yes	2104 (13.56)	115 (20.76)			148 (26.71)	115 (20.76)		
No	13072 (84.23)	417 (75.27)			390 (70.4)	417 (75.27)		
Borderline	343 (2.21)	22 (3.97)			16 (2.89)	22 (3.97)		
Cigarettes			7.88	<0.01			0.98	0.32
Yes	8475 (54.61)	336 (60.65)			352 (63.54)	336 (60.65)		
No	7044 (45.39)	218 (39.35)			202 (36.46)	218 (39.35)		
	***Mean ± SD***	***Mean ± SD***			***Mean ± SD***	***Mean ± SD***		
BMI (kg/m^2^)	28.49 ± 5.63	27.94 ± 5.1	2.28	0.02	28.12 ± 5.02	27.94 ± 5.1	0.6	0.55
Energy (kcal)	2314.58 ± 816.52	2016.87 ± 658.53	8.48	<0.01	1970.28 ± 673.56	2016.87 ± 658.53	-1.16	0.24
Protein (g)	91.65 ± 35.88	79.76 ± 30	7.71	<0.01	78.34 ± 28.92	79.76 ± 30	-0.8	0.42
Carbohydrate (g)	276.38 ± 108.11	243.94 ± 86.94	6.98	<0.01	237.49 ± 88.87	243.94 ± 86.94	-1.22	0.22
Total fat (g)	87.64 ± 38.57	77.95 ± 32.95	5.84	<0.01	76.55 ± 33.12	77.95 ± 32.95	-0.71	0.48
Total polyunsaturated fat (g)	19.43 ± 9.97	17.38 ± 8.56	4.77	<0.01	17.24 ± 8.48	17.38 ± 8.56	-0.27	0.79
Cholesterol (mg)	335.16 ± 205.23	307.63 ± 189.75	3.11	<0.01	301.88 ± 184.15	307.63 ± 189.75	-0.51	0.61
VitaminE (μg)	8.45 ± 5.39	8.25 ± 5.16	0.87	0.38	7.66 ± 4.51	8.25 ± 5.16	-2.01	0.04
VitaminA (μg)	646.12 ± 589.41	750.62 ± 524.42	-4.12	<0.01	707.73 ± 569.54	750.62 ± 524.42	-1.3	0.19
Calcium (mg)	981.68 ± 518.79	903.03 ± 409.87	3.53	<0.01	874.45 ± 476.25	903.03 ± 409.87	-1.07	0.28
Magnesium (mg)	318.35 ± 133.15	302.07 ± 117.54	2.84	<0.01	293.27 ± 123.92	302.07 ± 117.54	-1.21	0.23
Selenium (mg)	126.38 ± 53.22	110.56 ± 47.66	6.9	<0.01	108.04 ± 42.39	110.56 ± 47.66	-0.93	0.35
Caffeine (mg)	164.28 ± 194.84	142.64 ± 141.48	2.59	<0.01	166.75 ± 187.03	142.64 ± 141.48	2.42	0.02
Alcohol (g)	11.5 ± 25.27	7.48 ± 17.42	3.71	<0.01	6.84 ± 17.58	7.48 ± 17.42	-0.61	0.54

NHANES: National Health and Nutrition Examination Survey. PCa: prostate cancer. BMI: body mass index.

*GWAS summary statistics of PCa*

Summary-level genetic data of GWASs for PCa (diagnosed using ICD10 or ICD9 codes) were obtained from 3 sources: 1) the FinnGen research project, which included 6311 PCa cases and 74685 controls (GWAS ID=finn-b-C3_PROSTATE_EXALLC); 2) UK Biobank, with 9132 PCa cases and 173493 controls (GWAS ID=ieu-b-4809); and 3) the Prostate Cancer Association Group to Investigate Cancer-Associated Alterations in the Genome (PRACTICAL) consortium, with 79148 PCa cases and 61106 controls (GWAS ID=ieu-b-85)^13^.

### Statistical analysis

All analyses were restricted to male subjects. For NHANES data, we compared the distribution of basic information between PCa cases and non-PCa controls using the independent-sample t-test and the Pearson chi-squared test, as appropriate. Binary logistic regression was then used to evaluate the association between smoking and PCa. Four models were used in this analysis: 1) univariate logistic regression model containing only the smoking status; 2) Model 1: multivariate logistic regression containing the smoking status, with age, race, BMI, education level, hypertension status, and diabetes status as confounding factors; 3) Model 2: multivariable model 1 with dietary factors (as continuous variables) added as additional covariates; and 4) Model 3: multivariable model 1 with dietary factors (as categorizations as approximately determined using quartile distributions) added as additional covariates.

Propensity-score matching (PSM) was used to reduce selection bias by matching age, race, BMI, and education-level distributions as clinically pertinent between PCa cases and non-PCa controls. Matching was performed based on the nearest-neighbor method in a 1:1 ratio, and the balance after PSM was assessed using a histogram. Then, the above four models established by conditional logistic regression were analyzed using the matched sample.

The assumptions for the MR analysis are shown in Supplementary file Figure 1. The random-effects inverse-variance weighting (IVW) method was used as the main statistical model to estimate the associations between smoking behavior and PCa risk. Heterogeneity between the SNPs was evaluated by calculating Cochrane’s Q statistic and was considered to be presented when the Cochrane-Q-derived p<0.05. The F statistic (F=β^2^/SE^2^) was calculated to measure the instrument’s strength in the analyses, given a probable overlap between exposure and outcome data in the UK Biobank study. SNPs with F statistic <10 were excluded. Horizontal pleiotropy was detected using the MR-PRESSO analysis method, with a p<0.05 indicating its presence. The MR-PRESSO outlier test was performed when horizontal pleiotropy was detected. We then analyzed whether the MR results changed after removing outliers. Estimates from PRACTICAL, FinnGen, and UK biobank were combined using fixed-effects (I^2^<50%) and random-effects (I^2^≥50%) meta-analysis methods, as appropriate. Two other sensitivity analysis methods (weighted median and MR-Egger regression) were performed to assess the robustness of the MR results. The weighted median model can provide consistent estimates on the condition that ≥50% of the weight in the analysis comes from valid instrumental variables. The MR–Egger sensitivity estimator can provide unbiased estimates of causal effects, even if all SNPs in an instrument are invalid because of pleiotropy. However, it is necessary to satisfy the hypothesis that the effect of genetic variation pleiotropy on outcomes is independent of the effect of genetic variation on exposure factors (InSide). All analyses were two-sided, using odds ratio (OR) and 95% confidence interval (95% CI) to present associations. The analyses were performed using the TwoSampleMR and MR-PRESSO packages in R software (version 4.0.2).

## RESULTS

### Relationship between smoking and PCa risk in NHANES

**Figure 1:**
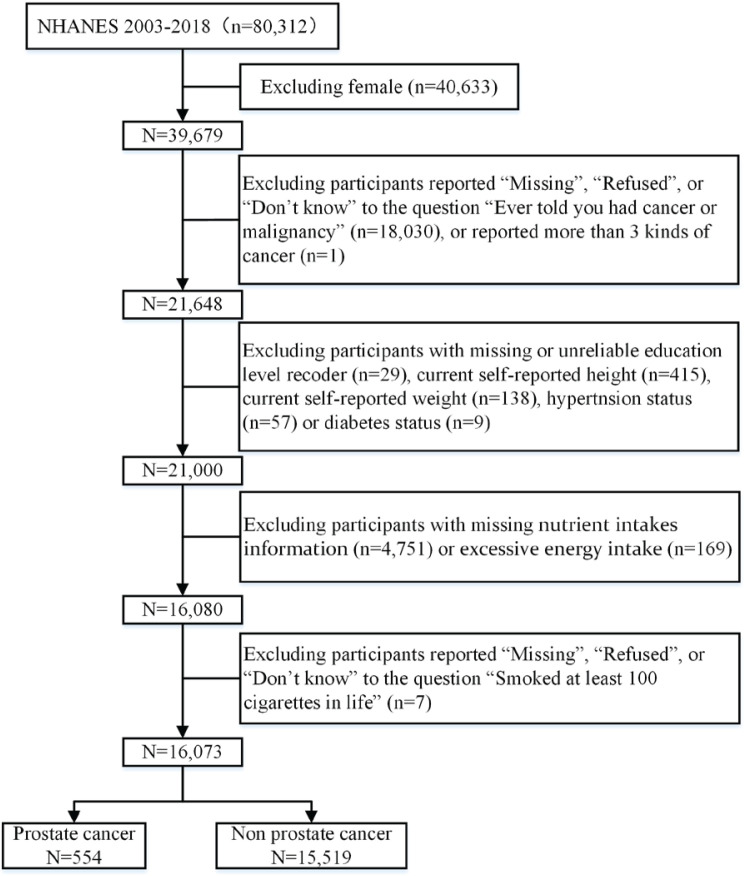
Flow chart of eligible participants selection for the cross-sectional study from the National Health and Nutrition Survey 2003–2018 before and after propensity-score matching (N=16073)

The final analysis was applied to 16073 participants, comprising 554 with PCa and 15519 without PCa. The data extraction process is shown in Figure 1. The distributions of basic information and dietary data between PCa and non-PCa participants are presented in Table 1. Relative to the non-PCa participants, PCa participants were generally older; comprised a larger proportion of non-Hispanic Whites and non-Hispanic Blacks; had higher prevalence rates of smoking, hypertension, and diabetes; and had a lower BMI and lower levels of all nutrient intakes except vitamin A. After PSM, 554 matched pairs were identified. The histogram (Supplementary file Figure 2) indicated that the balance between PCa and non-PCa participants is good. After PSM, the distribution of all factors was not significantly different except for PCa participants having a higher vitamin E and lower caffeine intake (Table 1).

**Figure 2:**
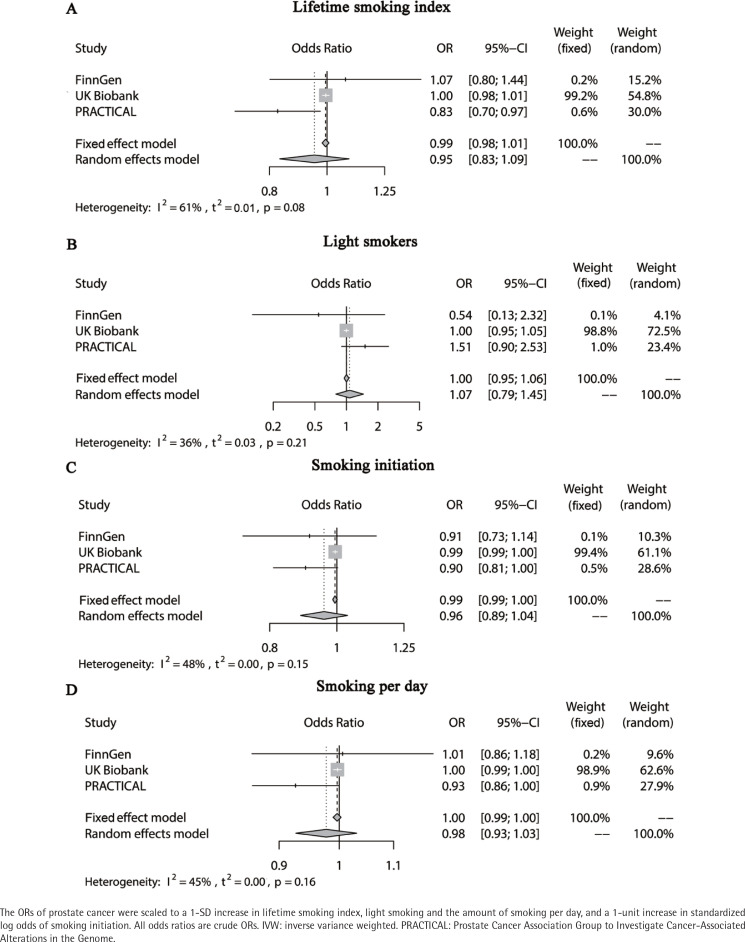
Forest plot of Mendelian randomization for associations of lifetime smoking index, light smoking, smoking initiation, and the amount of smoking per day with prostate cancer using the random-effects IVW method

The results from the analyses of the four logistic regression models using the population before and after PSM are presented in Table 2. None of the models showed a significant relationship between smoking status and PCa risk [adjusted OR with 95% CI before PSM=1.14 (0.95–1.37), 1.133 (0.94–1.37), 1.17 (0.96–1.41) for the three multivariable models; adjusted OR with 95% CI after PSM=1.15 (0.9–2.46), 1.12 (0.86–2.37), 1.14 (0.87–2.39) for the three multivariable models] with the exception of univariate logistic regression before PSM suggesting that non-smoking is a protective factor for PCa [OR with 95% CI: 0.78 (0.66–0.93), 1.13 (0.89–2.43) for univariate logistic regression before and after PSM].

**Table 2:** The association between smoking and PCa risk evaluated using logistic regressions for population from the National Health and Nutrition Examination Survey 2003–2018 before and after propensity-score matching (N=16073 before matching and N=1108 after matching)

*Models*	*Before propensity-score matching ^a^*		*After propensity-score matching ^b^*	
	*p*	*AOR (95% CI)*	*p*	*AOR (95% CI)*
Univariable model	0.01	0.78 (0.66–0.93)^c^	0.32	1.13 (0.89–2.43)^c^
Multivariable Model 1	0.17	1.14 (0.95–1.37)	0.26	1.15 (0.9–2.46)
Multivariable Model 2	0.2	1.133 (0.94–1.37)	0.39	1.12 (0.86–2.37)
Multivariable Model 3	0.11	1.17 (0.96–1.41)	0.34	1.14 (0.87–2.39)

Univariable model: univariate logistic regression containing only the smoking status. AOR: adjusted odds ratio. Model 1: multivariate logistic regression containing the smoking status, with age, race, BMI, education level, hypertension status, and diabetes status as confounding factors. Model 2: adding dietary factors (as continuous variables) as additional covariates to Model 1. Model 3: adding the dietary factors (as categorizations as approximately determined using the quartile distributions) as additional covariates to the Model 1. In the models, the exposure factor was smoking with the definition of smoking at least 100 cigarettes during life; the outcome was prostate cancer, defined as ever being told to have prostate cancer. NHANES: National Health and Nutrition Examination Survey. PCa: prostate cancer.

a Before propensity-score matching, the binary logistic regression was implemented.

b After propensity-score matching, the conditional logistic regression was implemented.

c The OR was crude odds ratio.

### MR analysis of the association of the lifetime smoking index with PCa

The lifetime smoking index was associated with 126 SNPs, and their F statistics ranged from 21.78 to 196. The genetically predicted lifetime smoking index was not associated with the risk of PCa in the FinnGen consortium or UK Biobank study. In contrast, it was negatively correlated with the risk of PCa in the PRACTICAL study (OR=0.83; 95% CI: 0.70–0.97). A meta-analysis of the three data sources indicated that there was no significant association (OR=0.95; 95% CI: 0.83–1.09) (Figure 2), and this result remained consistent in sensitivity analyses (weighted median and MR-Egger regression methods) (Supplementary file Figures 3 and 4). We detected significant heterogeneity in the UK Biobank study (Q=210.20, p=2.04×10^–7^) or PRACTICAL study (Q=256.59, p=1.24×10^–12^) but not in the FinnGen consortium (Q=107.36, p=0.73). MR-PRESSO analyses revealed significant horizontal pleiotropy for the UK Biobank and PRACTICAL studies (p<0.01), with one and three outliers found, respectively. The results did not change after removing the outliers (Supplementary file Table 2).

### MR analysis of the association of light smoking with PCa

There were three SNPs associated with light smoking, and their F statistics ranged from 31.76 to 160.15. Genetically predicted light smoking was not associated with PCa in the three PCa GWAS data sets. A meta-analysis of the three data sources indicated no significant association (OR=1.00; 95% CI: 0.95–1.06), and this result remained consistent in sensitivity analyses (Supplementary file Figures 3 and 4). We did not detect any heterogeneity in the UK Biobank study (Q=2.62, p=0.27), FinnGen consortium (Q=2.19, p=0.33), or PRACTICAL study (Q=0.28, p=0.87). MR-PRESSO analyses were not performed because of the small number of SNPs.

### MR analysis of the association of smoking initiation with PCa

Smoking initiation was associated with 92 SNPs, with F statistics ranging from 29.81 to 144.74. The MR results showed that smoking initiation was not associated with the risk of PCa in the three PCa GWAS data sets. A meta-analysis of the three data sources indicated no significant association (OR=0.99; 95% CI: 0.99–1.00), and this result remained consistent in sensitivity analyses (Supplementary file Figures 3 and 4). We detected significant heterogeneity in the UK Biobank study (Q=109.35, p=0.03) and PRACTICAL study (Q=170.16, p=8.44×10^–8^) but not in the FinnGen consortium (Q=104.92, p=0.05). MR-PRESSO revealed significant horizontal pleiotropy for the UK Biobank study (p=0.04) and PRACTICAL study (p<0.01), with zero and three outliers found, respectively. The results did not change after removing the outliers (Supplementary file Table 2).

### MR analysis of the association of the amount of smoking per day with PCa

The lifetime smoking index was associated with 23 SNPs, with F statistics ranging from 29.9 to 953.27. The genetically predicted amount of smoking per day was not associated with the risk of PCa in the FinnGen consortium or UK Biobank study. At the same time, it was negatively correlated with the PCa risk in the PRACTICAL study (OR=0.93; 95% CI: 0.86–1.00). A meta-analysis of the three data sources found no significant association (OR=1.00; 95% CI: 0.99–1.00), and this result remained consistent in sensitivity analyses (Supplementary Figures 3 and 4). We detected significant heterogeneity in the UK Biobank study (Q=36.62, p=0.02) but not in the FinnGen consortium (Q=19.80, p=0.53) or PRACTICAL study (Q=31.31, p=0.07). MR-PRESSO analyses revealed significant horizontal pleiotropy for the UK Biobank study (p=0.04), and no outliers were found.

## DISCUSSION

Reducing the serious disease burden of PCa worldwide^14^ requires modifiable risk factors to be identified, among which smoking has been widely investigated^1^. However, the research findings for this factor are inconsistent and still need clarification. The present observational study analyzed a large sample population in NHANES and performed an MR study using publicly available GWAS data, with both investigations indicating that smoking is unlikely to be associated with the risk of PCa.

The unclear findings from observational studies of the relationship between smoking and the risk of PCa^15,16^ are at least partially attributable not only to the study design and confounding factors but also to how smoking is defined and the proportions of subjects in different stages of PCa. Smoking is a lifestyle behavior that itself can be categorized into several states, such as current smoking, quitting smoking, severe smoking, mild smoking, and the use of filtered or unfiltered tobacco^17^. It is challenging to investigate factors with such high variability. Studies have shown that risk factors accumulate in the body to impact the disease^18^. The large heterogeneity of included populations can make it difficult to draw definitive conclusions, such as whether or not smoking impacts disease susceptibility. The main reason for choosing ‘smoked at least 100 cigarettes during the lifetime’ as one of the smoking statuses in the present study was due to this factor being relatively objective in NHANES and since this definition is less affected by the confounding effects caused by mild smoking. However, there is no clinical staging of PCa in NHANES, which makes it difficult to determine the impact of smoking on the risk of PCa at different stages.

The findings of this study support that smoking is not associated with the overall PCa risk. This is consistent with the results of most previous studies^1^, while there were also some publications with different conclusions. A meta-analysis pooling data from prospective cohorts provided evidence for a negative correlation between smoking and PCa incidence^19^. However, that analysis did not address the presence of heterogeneity in the merged results, nor were subgroup analyses conducted based on population race. As mentioned above, smoking is a behavior that has several states, and these may vary markedly with race or income level; for example, unfiltered tobacco accounts for a higher proportion of use in low- and middle-income countries. However, the meta-analysis conducted by Cirne et al.^20^ revealed that smoking is not associated with the risk of PCa in low- and middle-income countries. As expected, there are also study results suggesting that smoking is associated with a higher risk of PCa^2^. Perhaps even more informative is the meta-analysis by Islami et al.^3^ that produced mixed results for the association between cigarette smoking and PCa risk, with their overall analysis of all included studies showing no or negative correlations. In contrast, those studies completed up to 1995 showed a positive correlation^3^. Those authors attributed this to smoking reducing the risk of inert non-invasive cancer, which has dominated in recent years while promoting more-invasive cancer. Another analysis based on biopsy data validated that result by finding that all cases of PCa as well as only those of low-grade PCa were not significantly associated with current or past smoking, while it was associated with an increased risk of high-grade PCa^21^. These results further indicate that the relationship between smoking and PCa risk is influenced by the date range of the analyzed data, which is mainly attributable to differences in the risk grading of PCa patients associated with prostate-specific antigen (PSA) screening policies^22^. In short, the relationship between smoking and PCa risk is very complex and cannot be simply explained by the theory that harmful substances such as nicotine in tobacco increase the risk of cancer^23^.

Any population-based study analyzing the relationship between smoking and PCa risk is complicated by susceptibility to various confounding factors. Thus, we conducted an MR study whose results also supported those from the NHANES-based investigation that there was no evidence that smoking was associated with the risk of PCa. Larsson and Burgess^24^ conducted similar MR research and unexpectedly found a statistically non-significant negative correlation between smoking initiation and PCa. We speculate that these different conclusions are mainly attributable to discrepancies in the instrumental variables. We applied stricter inclusion criteria and found only 92 SNPs related to smoking initiation, which is far fewer than the 378 found by Larsson and Burgess^24^. Including more instrumental variables will generally increase the probability of horizontal pleiotropy being present, which will lead to the instrumental variables not exhibiting exclusivity and therefore causing parameter estimation errors^25^; however, the status of horizontal pleiotropy was not reported in the study of Larsson and Burgess^24^. In addition, those authors obtained significant results using the PRACTICAL population. Similar to this, our study found that the lifetime smoking index and the amount of smoking per day were negatively correlated with the risk of PCa in the PRACTICAL population but not in the other two databases (UK Biobank and the Finland-based FinnGen consortium). We speculate that differences in sample sizes cause the inconsistent results between different database populations, while the negative correlation between smoking and PCa reflects detection bias; that is, the control group may be contaminated, especially among smokers^26^, since smokers may be less likely to undergo PSA screening and therefore less likely to be diagnosed with early-stage PCa.

The results from the two parts of this study were relatively stable. In our NHANES-based investigation, we matched important risk factors for PCa such as age, race, and BMI, which did not change the results. For the MR analysis, the weighting of the UK Biobank research was very high (>98%) in the meta-analysis, possibly due to this being the largest sample, which therefore exerted the dominant effect on the merged results. The results of our MR study using UK Biobank population were relatively reliable. There are two aspects to note: 1) all instrumental variables other than the SNP for light smoking (derived from UK Biobank) were all derived from GWAS and the Sequencing Consortium of Alcohol and Nicotine use, which resulted in very low sample-overlap of exposure factors and the results; and 2) although horizontal pleiotropy was present in the MR results for the UK Biobank and PRACTICAL populations, the MR results did not change when outliers were excluded.

### Limitations

This study has several limitations. Firstly, PCa was not classified into different stages, and studies have shown that the association between smoking and PCa is mainly present in patients with invasive PCa^27^. The outcomes of the present study come from different GWAS populations. Although advanced PCa accounts for 19.15% of those in the PRACTICAL population^28^, the proportions of the other two databases are not publicly known, and relevant information cannot be obtained from NHANES. We, therefore, did not analyze the relationship between smoking and invasive PCa. Secondly, the definition of smoking can introduce limitations. We selected one variable in NHANES and four smoking variables in the MR analysis to represent the smoking status. The definition of smoking used in this study may not fully reflect the multiple states of smoking behavior in the included populations. While smoking may exert harmful effects on physical health, mainly via substances such as nicotine, its direct impact may be on the respiratory system^29^. However, even if there is such an impact on PCa, both previous studies and the present study indicate that the level of evidence is much weaker than that for non-modifiable risk factors such as age^30^. Thirdly, despite applying strict inclusion criteria for the instrumental variables, heterogeneity remained significant in some present analyses. Although we used the random-effects model for IVW, future studies that apply stratified analyses are required. Fourthly, unobserved pleiotropy cannot be addressed in MR analysis. Fifthly, for smoking status, whether the observed associations differ by age and other potential factors, and by PCa severity, could not be examined based on summary-level data in this study.

## CONCLUSIONS

This study, using NHANES data and MR analysis, found no evidence of an association between smoking and the risk of PCa. Further studies with larger samples are needed to determine if there are any associations between other forms of smoking and the risk of PCa at different stages.

## Supplementary Material



## Data Availability

The datasets used and/or analyzed during the current study are available from the website of NHANES (https://www.cdc.gov/nchs/nhanes/) and IEU OpenGWAS project (https://gwas.mrcieu.ac.uk/datasets/).

## References

[1] Bergengren O, Pekala KR, Matsoukas K, et al. 2022 update on prostate cancer epidemiology and risk factors-a systematic review. Eur Urol. 2023;84(2):191-206. doi:10.1016/j.eururo.2023.04.021 PMC10851915 37202314

[2] Bashir MN, Ahmad MR, Malik A. Risk factors of prostate cancer: a case-control study in Faisalabad, Pakistan. Asian Pac J Cancer Prev. 2014;15(23):10237-10240. doi:10.7314/apjcp.2014.15.23.10237 25556453

[3] Islami F, Moreira DM, Boffetta P, Freedland SJ. A systematic review and meta-analysis of tobacco use and prostate cancer mortality and incidence in prospective cohort studies. Eur Urol. 2014;66(6):1054-1064. doi:10.1016/j.eururo.2014.08.059 25242554 PMC4566150

[4] Larsson SC, Carter P, Kar S, et al. Smoking, alcohol consumption, and cancer: a mendelian randomisation study in UK Biobank and international genetic consortia participants. PLoS Med. 2020;17(7):e1003178. doi:10.1371/journal.pmed.1003178 32701947 PMC7377370

[5] Johnson CL, Dohrmann SM, Burt VL, Mohadjer LK. National health and nutrition examination survey: sample design, 2011-2014. Vital Health Stat 2. 2014(162):1-33. 25569458

[6] U.S. Centers for Disease Control and Prevention. NHANES -National Health and Nutrition Examination Survey. Accessed May 16, 2024. https://www.cdc.gov/nchs/nhanes/

[7] Smith GD, Ebrahim S. ‘Mendelian randomization’: can genetic epidemiology contribute to understanding environmental determinants of disease?. Int J Epidemiol. 2003;32(1):1-22. doi:10.1093/ije/dyg070 12689998

[8] Wang M, Jian Z, Yuan C, Jin X, Li H, Wang K. Coffee consumption and prostate cancer risk: results from National Health and Nutrition Examination Survey 1999-2010 and Mendelian Randomization Analyses. Nutrients. 2021;13(7):2317. doi:10.3390/nu13072317 34371827 PMC8308488

[9] Larsson SC, Burgess S. Appraising the causal role of smoking in multiple diseases: a systematic review and meta-analysis of Mendelian randomization studies. EBioMedicine. 2022;82:104154. doi:10.1016/j.ebiom.2022.104154 35816897 PMC9278068

[10] Sen A, Papadimitriou N, Lagiou P, et al. Coffee and tea consumption and risk of prostate cancer in the European Prospective Investigation into Cancer and Nutrition. Int J Cancer. 2019;144(2):240-250. doi:10.1002/ijc.31634 29943826

[11] Vinceti M, Filippini T, Cilloni S, Crespi CM. The epidemiology of selenium and human cancer. Adv Cancer Res. 2017;136:1-48. doi:10.1016/bs.acr.2017.07.001 29054414

[12] Wootton RE, Richmond RC, Stuijfzand BG, et al. Evidence for causal effects of lifetime smoking on risk for depression and schizophrenia: a Mendelian randomisation study. Psychol Med. 2020;50(14):2435-2443. doi:10.1017/S0033291719002678 31689377 PMC7610182

[13] Liu M, Jiang Y, Wedow R, et al. Association studies of up to 1.2 million individuals yield new insights into the genetic etiology of tobacco and alcohol use. Nat Genet. 2019;51(2):237-244. doi:10.1038/s41588-018-0307-5 30643251 PMC6358542

[14] IEU OpenGWAS project. GWAS summary data. Accessed May 16, 2024. https://gwas.mrcieu.ac.uk/

[15] Seibert TM, Garraway IP, Plym A, et al. Genetic risk prediction for prostate cancer: implications for early detection and prevention. Eur Urol. 2023;83(3):241-248. doi:10.1016/j.eururo.2022.12.021 36609003

[16] Pernar CH, Ebot EM, Wilson KM, Mucci LA. The epidemiology of prostate cancer. Cold Spring Harb Perspect Med. 2018;8(12):a030361. doi:10.1101/cshperspect.a030361 29311132 PMC6280714

[17] De Nunzio C, Andriole GL, Thompson IM Jr, Freedland SJ. Smoking and prostate cancer: a systematic review. Eur Urol Focus. 2015;1(1):28-38. doi:10.1016/j.euf.2014.10.002 28723351

[18] Wipfli H, Samet JM. One hundred years in the making: the global tobacco epidemic. Annu Rev Public Health. 2016;37:149-166. doi:10.1146/annurev-publhealth-032315-021850 26772406

[19] Siqueira LM; Committee on Substance Use and Prevention. Nicotine and tobacco as substances of abuse in children and adolescents. Pediatrics. 2017;139(1):e20163436. doi:10.1542/peds.2016-3436 27994114

[20] Al-Fayez S, El-Metwally A. Cigarette smoking and prostate cancer: a systematic review and meta-analysis of prospective cohort studies. Tob Induc Dis. 2023;21:19. doi:10.18332/tid/157231 36762260 PMC9900478

[21] Cirne F, Kappel C, Zhou S, et al. Modifiable risk factors for prostate cancer in low- and lower-middle-income countries: a systematic review and meta-analysis. Prostate Cancer Prostatic Dis. 2022;25(3):453-462. doi:10.1038/s41391-022-00570-1 35790786

[22] Ho T, Howard LE, Vidal AC, et al. Smoking and risk of low- and high-grade prostate cancer: results from the REDUCE study. Clin Cancer Res. 2014;20(20):5331-5338. doi:10.1158/1078-0432.CCR-13-2394 25139338 PMC4199866

[23] Desai MM, Cacciamani GE, Gill K, et al. Trends in incidence of metastatic prostate cancer in the US. JAMA Netw Open. 2022;5(3):e222246. doi:10.1001/jamanetworkopen.2022.2246 35285916 PMC9907338

[24] Price SN, Studts JL, Hamann HA. Tobacco use assessment and treatment in cancer patients: a scoping review of oncology care clinician adherence to clinical practice guidelines in the U.S. Oncologist. 2019;24(2):229-238. doi:10.1634/theoncologist.2018-0246 30446582 PMC6369951

[25] Carter AR, Sanderson E, Hammerton G, et al. Mendelian randomisation for mediation analysis: current methods and challenges for implementation. Eur J Epidemiol. 2021;36(5):465-478. doi:10.1007/s10654-021-00757-1 33961203 PMC8159796

[26] He H, Liu T, Zhao F, Feng X, Lyu J, Gao Y. Nonlinear relationship between age and likelihood of undergoing prostate-specific antigen testing, and the predictive factors of testing at different ages. Am J Mens Health. 2021;15(3):15579883211026515. doi:10.1177/15579883211026515 34167355 PMC8246524

[27] Zu K, Giovannucci E. Smoking and aggressive prostate cancer: a review of the epidemiologic evidence. Cancer Causes Control. 2009;20(10):1799-1810. doi:10.1007/s10552-009-9387-y 19562492

[28] Bouras E, Karhunen V, Gill D, et al. Circulating inflammatory cytokines and risk of five cancers: a Mendelian randomization analysis. BMC Med. 2022;20(1):3. doi:10.1186/s12916-021-02193-0 35012533 PMC8750876

[29] He H, Pan Z, Wu J, Hu C, Bai L, Lyu J. Health effects of tobacco at the global, regional, and national levels: results from the 2019 Global Burden of Disease Study. Nicotine Tob Res. 2022;24(6):864-870. doi:10.1093/ntr/ntab265 34928373

[30] American Cancer Society. Prostate Cancer Risk Factors. Accessed May 16, 2024. https://www.cancer.org/cancer/types/prostate-cancer/causes-risks-prevention/risk-factors.html

