# Supplementary material for: Effect of smoking on prostate cancer: Results from the National Health and Nutrition Examination Survey 2003–2018 and Mendelian randomization analyses
Source: Tob Induc Dis. 2024 Jun 4;22:10.18332/tid/189199. doi: 10.18332/tid/189199 (PMC11149497; doi:10.18332/tid/189199)
Supplement: Supplementary file 1 [file TID-22-100-s1.pdf]

Effect of smoking on prostate cancer: results from the National Health and Nutrition Examination Survey 2003–2018 and Mendelian randomization analyses

Contents:

Supplementary Table 1. The information of Single-nucleotide polymorphisms associated with smoking behaviors and their associations with outcomes.

Supplementary Figure 1. The assumptions of MR analysis.

Assumption 1, the genetic variants proposed as instrumental variables should be robustly associated with the risk factor of interest; Assumption 2, the used genetic variants should not be associated with potential confounders; Assumption 3, the selected genetic variants should affect the risk of the outcome merely through the risk factor, not via alternative pathways. MR, Mendelian randomization; SNPs, single-nucleotide polymorphisms; LD, linkage disequilibrium; IVW, inverse-variance weighted; MR-PRESSO, MR pleiotropy residual sum and outlier; PCa, prostate cancer; GWAS, genome-wide association study; PRACTICA, Prostate Cancer Association Group to Investigate Cancer-Associated Alterations in the Genome.

Supplementary Figure 2. Distribution of propensity scores of PCa and non PCa groups before and after matching. Before matching, the distribution of propensity score between non PCa and PCa was obviously different, while after matching, the distribution was similar.

Supplementary Figure 3. Forest plot of Mendelian randomization for associations of lifetime smoking index, light smoking, smoking initiation and the amount of smoking per day with

prostate cancer using the weighted median method. The ORs of prostate cancer were scaled to a 1-SD increase in lifetime smoking index, light smoking and the amount of smoking per day, and a 1-unit increase in standardized log odds of smoking initiation. All of ORs were crude ORs. OR, odds ratio; CI, confidence interval; PRACTICAL, Prostate Cancer Association Group to Investigate Cancer-Associated Alterations in the Genome.

Supplementary Figure 4. Forest plot of Mendelian randomization for associations of lifetime smoking index, light smoking, smoking initiation and the amount of smoking per day with prostate cancer using the MR-Egger regression method. The ORs of prostate cancer were scaled to a 1-SD increase in lifetime smoking index, light smoking and the amount of smoking per day, and a 1-unit increase in standardized log odds of smoking initiation. All of ORs were crude ORs. OR, odds ratio; CI, confidence interval; PRACTICAL, Prostate Cancer Association Group to Investigate Cancer-Associated Alterations in the Genome.

Supplementary Table 2. The results of pleiotropy test and MR-PRESSO.

Supplementary Table 1. The information of Single-nucleotide polymorphisms associated with smoking behaviors and their associations with outcomes.

exposure	SNPID	CHR	EA	OA	EAF	Associations with exposure			Associations with PCa (FinnGen)			Associations with PCa (UK Biobank)			Associations with PCa (PRACTICAL)		
						Beta	SE	P	Beta	SE	P	Beta	SE	P	Beta	SE	P
Lifetime smoking index	rs10052591	5	T	C	0.57	0.01	2E-03	2.1E-09	8.2E-03	2.17E-02	7.06E-01	-5.4E-04	7.29E-04	4.6E-01	1.1E-02	8.1E-03	1.77E-01
	rs10226228	7	A	G	0.63	-0.02	2E-03	2E-15	3.5E-03	2.35E-02	8.83E-01	-1.13E-03	7.48E-04	1.3E-01	9.6E-03	8.5E-03	2.57E-01
	rs10282292	7	C	T	0.36	0.01	2E-03	5.9E-10	1.08E-02	2.21E-02	6.26E-01	1.24E-03	7.52E-04	9.8E-02	7E-04	8.5E-03	9.3E-01
	rs1050847	16	C	T	0.43	0.01	2E-03	1.4E-08	6E-03	2.14E-02	7.78E-01	-1.04E-03	7.3E-04	1.6E-01	3.3E-03	8.4E-03	6.92E-01
	rs10823968	10	A	T	0.63	0.01	2E-03	2.1E-08	-7.00E-03	2.15E-02	7.45E-01	5.73E-04	7.57E-04	4.5E-01	9.3E-03	8.4E-03	2.66E-01
	rs10879871	12	T	G	0.34	-0.01	2E-03	5E-11	1.13E-02	2.23E-02	6.13E-01	-9.91E-04	7.58E-04	1.9E-01	-9.3E-03	8.5E-03	2.75E-01
	rs10918701	1	G	A	0.37	0.01	2E-03	2.1E-08	-7.2E-03	2.22E-02	7.45E-01	5.2E-04	7.46E-04	4.9E-01	6.4E-03	8.4E-03	4.46E-01
	rs10922907	1	A	T	0.45	0.02	2E-03	3E-13	-4.43E-02	2.19E-02	4.31E-02	3.59E-04	7.25E-04	6.2E-01	7.2E-03	8.6E-03	4.05E-01
	rs11210229	1	A	G	0.38	0.02	2E-03	2E-16	-3.8E-03	2.2E-02	8.64E-01	2.02E-04	7.41E-04	7.8E-01	-1.5E-03	8.1E-03	8.54E-01
	rs112282219	11	G	A	0.96	-0.03	5E-03	3.8E-11	-4.44E-02	6.68E-02	5.07E-01	-3.01E-03	1.82E-03	9.8E-02	-4.68E-02	2.2E-02	3.3E-02
	rs11255908	10	T	G	0.74	-0.02	2E-03	2.3E-10	-1.96E-02	2.89E-02	4.99E-01	5.24E-04	8.26E-04	5.3E-01	-2.03E-02	9.6E-03	3.45E-02
	rs113382419	9	C	A	0.89	-0.04	3E-03	3E-37	3.37E-02	5.88E-02	5.67E-01	-2.56E-03	1.15E-03	2.6E-02	-1.74E-02	1.39E-02	2.09E-01
	rs11768481	7	C	A	0.67	0.01	2E-03	9.9E-10	-2.55E-02	2.18E-02	2.42E-01	-8.16E-04	7.76E-04	2.9E-01	-1.7E-03	9.5E-03	8.54E-01
	rs11783093	8	C	T	0.84	0.02	3E-03	1.2E-16	-3.02E-02	3.22E-02	3.48E-01	-1.49E-03	9.84E-04	1.3E-01	-1.76E-02	1.14E-02	1.22E-01
	rs11861214	16	G	T	0.78	0.01	2E-03	2E-08	-1.06E-02	2.25E-02	6.36E-01	1.23E-03	8.77E-04	1.6E-01	9.8E-03	9.5E-03	3.04E-01
	rs1193237	1	G	C	0.44	-0.01	2E-03	2.8E-08	-2.59E-02	2.12E-02	2.23E-01	-7.08E-04	7.29E-04	3.3E-01	6.4E-03	8.7E-03	4.66E-01
	rs11948770	5	T	C	0.77	-0.02	2E-03	4.9E-10	-1.44E-02	2.42E-02	5.52E-01	1.13E-03	8.58E-04	1.9E-01	3.7E-03	9.6E-03	7.01E-01
	rs12202536	6	A	G	0.51	-0.01	2E-03	2.8E-09	3.31E-02	2.14E-02	1.22E-01	5.27E-04	7.21E-04	4.7E-01	-2E-04	8E-03	9.84E-01
	rs1221148	9	C	G	0.59	0.01	2E-03	7.3E-11	-3.57E-02	2.16E-02	9.77E-02	-7.4E-04	7.31E-04	3.1E-01	5.8E-03	8.8E-03	5.1E-01
	rs12244388	10	G	A	0.66	-0.02	2E-03	1.4E-19	-3.69E-02	2.2E-02	9.29E-02	-2.41E-03	7.62E-04	1.6E-03	-2.22E-02	8.4E-03	8.21E-03
	rs1246265	9	T	C	0.31	-0.01	2E-03	4.2E-09	-1.97E-02	2.37E-02	4.07E-01	-7.77E-06	7.84E-04	9.9E-01	2E-04	8.8E-03	9.83E-01

rs12481282	20	G	C	0.72	-0.01	2E-03	7.8E-09	-1.2E-02	2.38E-02	6.13E-01	-1.55E-03	8.06E-04	5.5E-02	3.9E-03	9.2E-03	6.74E-01
rs12623702	2	A	G	0.61	-0.01	2E-03	7.7E-12	3.12E-02	2.14E-02	1.46E-01	2.68E-04	7.43E-04	7.2E-01	5E-04	8.3E-03	9.48E-01
rs12708665	16	A	G	0.29	-0.01	2E-03	3.5E-09	-2.35E-02	2.38E-02	3.25E-01	1.35E-04	8E-04	8.7E-01	-1.08E-02	9E-03	2.29E-01
rs12831617	12	C	T	0.76	-0.01	2E-03	1.9E-08	-5.4E-03	2.88E-02	8.52E-01	2.31E-04	8.5E-04	7.9E-01	-1.86E-02	9.5E-03	5.06E-02
rs12967855	18	A	G	0.33	0.01	2E-03	3.1E-08	1.26E-02	2.4E-02	5.99E-01	8.31E-04	7.7E-04	2.8E-01	1E-04	8.8E-03	9.88E-01
rs13009008	2	A	G	0.33	0.01	2E-03	4.6E-09	9.9E-03	2.2E-02	6.53E-01	4.09E-04	7.67E-04	5.9E-01	1.01E-02	8.5E-03	2.32E-01
rs13016665	2	C	A	0.58	-0.01	2E-03	1.8E-09	9.1E-03	2.14E-02	6.69E-01	/	/	/	/	/	/
rs13153393	5	A	G	0.88	-0.02	3E-03	2.5E-10	-6.3E-03	3.68E-02	8.65E-01	-1.33E-03	1.13E-03	2.4E-01	-9.1E-03	1.35E-02	5E-01
rs13296519	9	G	T	0.61	-0.01	2E-03	8.1E-12	1.2E-02	2.19E-02	5.83E-01	1.49E-03	7.37E-04	4.4E-02	8.8E-03	8.6E-03	3.06E-01
rs136233	22	A	G	0.81	-0.01	3E-03	1.8E-08	2.91E-02	2.82E-02	3.01E-01	-3.61E-04	9.19E-04	6.9E-01	1.05E-02	1.09E-02	3.35E-01
rs147412694	21	G	A	0.85	-0.02	3E-03	2.9E-09	8E-03	3.15E-02	7.99E-01	-8.16E-04	1.02E-03	4.2E-01	2.5E-02	1.19E-02	3.64E-02
rs17309874	11	G	A	0.74	-0.02	2E-03	9.7E-13	-2.84E-02	2.36E-02	2.29E-01	-1.83E-03	8.23E-04	2.6E-02	-1.26E-02	9.3E-03	1.75E-01
rs17553262	10	A	C	0.89	-0.02	3E-03	5.3E-09	-5.5E-02	4.15E-02	1.86E-01	-6.72E-04	1.14E-03	5.5E-01	-1.16E-02	1.29E-02	3.67E-01
rs17576594	4	G	A	0.72	0.02	2E-03	1.7E-12	1.92E-02	2.34E-02	4.13E-01	-3.93E-04	8.08E-04	6.3E-01	1.22E-02	8.9E-03	1.7E-01
rs1922018	7	C	T	0.36	0.01	2E-03	3E-12	-4.05E-02	2.25E-02	7.27E-02	-1.02E-03	7.48E-04	1.7E-01	-2.13E-02	8.4E-03	1.09E-02
rs1931263	1	G	T	0.51	-0.01	2E-03	4E-08	4.6E-03	2.13E-02	8.3E-01	1.35E-04	7.22E-04	8.5E-01	-1.1E-03	8E-03	8.91E-01
rs1933270	1	T	G	0.36	0.01	2E-03	1.5E-10	5.48E-02	2.18E-02	1.2E-02	9.74E-04	7.46E-04	1.9E-01	1.1E-03	8.3E-03	8.93E-01
rs202645	22	A	G	0.20	-0.02	2E-03	3.9E-09	-1.18E-02	2.52E-02	6.4E-01	-8.02E-04	8.96E-04	3.7E-01	-2.53E-02	9.9E-03	1.05E-02
rs2062882	8	G	A	0.59	-0.01	2E-03	1.1E-08	-1.23E-02	2.14E-02	5.66E-01	/	/	/	/	/	/
rs2080870	5	A	T	0.26	0.01	2E-03	4.9E-08	-7E-04	2.48E-02	9.76E-01	-8.34E-04	8.23E-04	3.1E-01	-3E-04	9.2E-03	9.73E-01
rs2254710	6	C	A	0.24	0.01	2E-03	3.5E-08	-4.36E-02	2.27E-02	5.52E-02	7.14E-04	8.48E-04	4E-01	1.47E-02	9.3E-03	1.15E-01
rs2401924	7	G	C	0.50	0.02	2E-03	2.7E-14	7.9E-03	2.15E-02	7.12E-01	5.98E-04	7.22E-04	4.1E-01	-1.6E-03	8.4E-03	8.5E-01
rs245774	5	A	G	0.27	-0.01	2E-03	7.4E-09	-1.8E-02	2.27E-02	4.29E-01	-1.96E-04	8.1E-04	8.1E-01	1.59E-02	9E-03	7.68E-02
rs2675638	10	G	A	0.58	0.01	2E-03	1.3E-09	5.8E-03	2.2E-02	7.93E-01	-4.61E-04	7.28E-04	5.3E-01	3.2E-03	8.2E-03	6.99E-01
rs2678670	2	A	T	0.49	0.01	2E-03	3.1E-10	-2.58E-02	2.13E-02	2.25E-01	-2.94E-04	7.22E-04	6.8E-01	-9.6E-03	8.1E-03	2.33E-01
rs2838834	21	C	T	0.70	-0.01	2E-03	6.3E-10	-8.5E-03	2.46E-02	7.28E-01	-8.4E-05	7.87E-04	9.1E-01	-4.1E-03	8.7E-03	6.43E-01

rs28485305	15	C	T	0.63	0.01	2E-03	2.6E-08	-2.87E-02	2.19E-02	1.9E-01	7.69E-04	7.49E-04	3E-01	-1.79E-02	8.3E-03	3.11E-02
rs2867112	2	T	G	0.84	0.02	3E-03	4.8E-15	-3.15E-02	2.88E-02	2.73E-01	1.95E-03	9.82E-04	4.7E-02	2.94E-02	1.05E-02	5.28E-03
rs2890772	2	G	T	0.41	-0.02	2E-03	2.1E-22	5.6E-03	2.45E-02	8.2E-01	1.43E-03	7.32E-04	5.1E-02	8.5E-03	8.4E-03	3.11E-01
rs2894808	6	T	A	0.92	-0.02	4E-03	3.5E-09	-4.86E-02	4.54E-02	2.84E-01	-3.59E-03	1.35E-03	7.9E-03	5.4E-03	1.6E-02	7.36E-01
rs317021	4	T	A	0.81	-0.02	3E-03	1.1E-10	4.75E-02	2.75E-02	8.4E-02	-8.51E-04	9.3E-04	3.6E-01	8E-04	1.13E-02	9.41E-01
rs326341	3	G	A	0.53	0.01	2E-03	1.2E-11	-1.01E-02	2.15E-02	6.38E-01	8.72E-04	7.24E-04	2.3E-01	-1.9E-03	8.2E-03	8.14E-01
rs329120	5	C	T	0.58	0.01	2E-03	6.3E-12	2.01E-02	2.14E-02	3.49E-01	1.8E-03	7.31E-04	1.4E-02	2.87E-02	8E-03	3.26E-04
rs34866095	11	A	G	0.69	-0.01	2E-03	1.2E-08	1.89E-02	2.48E-02	4.45E-01	-8.87E-04	7.84E-04	2.6E-01	8E-04	8.7E-03	9.29E-01
rs348809	20	A	G	0.35	-0.01	2E-03	1.3E-08	1.8E-03	2.29E-02	9.37E-01	-4.62E-04	7.57E-04	5.4E-01	-9.4E-03	8.9E-03	2.93E-01
rs35169606	8	T	G	0.61	0.01	2E-03	1.2E-09	1.76E-02	3.2E-02	5.83E-01	-6.13E-04	7.51E-04	4.1E-01	-1.99E-02	8.5E-03	1.92E-02
rs35175834	15	G	A	0.79	-0.02	2E-03	4.6E-22	6.7E-03	2.55E-02	7.92E-01	-4.14E-06	8.84E-04	1E+00	-1.62E-02	9.6E-03	9.16E-02
rs35343344	19	C	A	0.73	0.01	2E-03	8.8E-09	-4.17E-02	2.5E-02	9.55E-02	5.66E-04	8.3E-04	5E-01	1.33E-02	9.5E-03	1.63E-01
rs359243	2	T	C	0.39	-0.01	2E-03	9.5E-10	-1E-04	2.22E-02	9.97E-01	-6.7E-04	7.41E-04	3.7E-01	1.44E-02	8.3E-03	8.3E-02
rs369230	16	G	T	0.31	-0.01	2E-03	1.8E-09	-1.02E-02	2.24E-02	6.5E-01	1.09E-03	7.87E-04	1.7E-01	-2.1E-03	9.1E-03	8.2E-01
rs3742365	14	T	C	0.60	-0.02	2E-03	2.5E-14	1.65E-02	2.15E-02	4.45E-01	2.3E-03	7.37E-04	1.8E-03	2.17E-02	8.1E-03	7.69E-03
rs3769949	2	T	A	0.53	-0.01	2E-03	2.5E-09	-3.95E-02	2.36E-02	9.48E-02	2.72E-05	7.21E-04	9.7E-01	4.3E-03	8.3E-03	5.99E-01
rs3811038	2	T	C	0.72	-0.01	2E-03	8.9E-10	-2.03E-02	2.54E-02	4.24E-01	2.14E-05	8.11E-04	9.8E-01	-8.9E-03	9E-03	3.24E-01
rs3896224	10	A	G	0.59	0.01	2E-03	1.1E-11	-2E-02	2.14E-02	3.49E-01	1.72E-04	7.38E-04	8.2E-01	7.9E-03	8.1E-03	3.32E-01
rs421983	3	T	C	0.52	0.01	2E-03	3.3E-10	2.25E-02	2.14E-02	2.94E-01	-1.76E-03	7.21E-04	1.5E-02	-1.35E-02	8.2E-03	1.02E-01
rs4391802	11	A	G	0.71	0.02	2E-03	1.4E-11	1.67E-02	2.49E-02	5.04E-01	-8.13E-04	7.94E-04	3.1E-01	-1.58E-02	9.3E-03	8.79E-02
rs4473348	2	A	T	0.25	-0.02	2E-03	6.4E-11	-3.4E-03	2.52E-02	8.94E-01	1.83E-03	8.31E-04	2.7E-02	1.89E-02	9.3E-03	4.19E-02
rs4543592	9	T	C	0.52	-0.01	2E-03	4.5E-10	-3E-04	2.18E-02	9.89E-01	-6.16E-04	7.22E-04	3.9E-01	-4.4E-03	8.1E-03	5.86E-01
rs4571506	5	C	T	0.54	0.01	2E-03	1.5E-08	-2.07E-02	2.14E-02	3.34E-01	1.68E-03	7.3E-04	2.1E-02	1.29E-02	8.1E-03	1.11E-01
rs4671357	2	T	C	0.52	-0.01	2E-03	1.1E-11	-1.09E-02	2.18E-02	6.18E-01	-1.54E-03	7.24E-04	3.3E-02	-1.37E-02	8.2E-03	9.59E-02
rs4731925	7	C	T	0.32	-0.01	2E-03	2.6E-08	-3.02E-02	2.34E-02	1.98E-01	-2.88E-04	7.75E-04	7.1E-01	1.09E-02	8.9E-03	2.2E-01
rs4814873	20	C	T	0.77	0.01	2E-03	2.9E-09	2.2E-03	2.48E-02	9.3E-01	1.19E-03	8.5E-04	1.6E-01	9E-03	9.5E-03	3.42E-01

rs4949465	1	T	C	0.87	-0.02	3E-03	1.7E-08	-2.74E-02	2.86E-02	3.39E-01	-9.11E-04	1.07E-03	4E-01	-5.3E-03	1.23E-02	6.66E-01
rs4957528	5	A	C	0.21	-0.02	2E-03	4.2E-09	3.5E-03	3.02E-02	9.08E-01	-8.27E-04	8.96E-04	3.6E-01	-4.9E-03	1.06E-02	6.45E-01
rs549845	1	G	A	0.30	0.02	2E-03	8.3E-14	1.82E-02	2.41E-02	4.5E-01	4.86E-04	7.86E-04	5.4E-01	2.7E-02	8.9E-03	2.31E-03
rs57611503	16	G	A	0.49	0.01	2E-03	4E-08	-1.2E-02	2.15E-02	5.77E-01	5.08E-05	7.34E-04	9.4E-01	6.5E-03	8.3E-03	4.38E-01
rs6011779	20	C	T	0.19	0.03	3E-03	2.3E-27	3.14E-02	2.5E-02	2.1E-01	4.29E-04	9.15E-04	6.4E-01	2.34E-02	1.05E-02	2.62E-02
rs60952428	16	T	C	0.91	0.02	3E-03	3E-08	6.6E-03	3.66E-02	8.56E-01	-1.06E-03	1.26E-03	4E-01	5.8E-03	1.41E-02	6.79E-01
rs6119897	20	G	A	0.76	-0.02	2E-03	3.6E-15	-2.5E-03	2.27E-02	9.12E-01	7.37E-04	8.49E-04	3.9E-01	1.6E-03	9.4E-03	8.66E-01
rs61796681	4	A	T	0.91	-0.02	4E-03	4.2E-08	-4.38E-02	3.64E-02	2.29E-01	1.76E-04	1.27E-03	8.9E-01	-8E-04	1.48E-02	9.54E-01
rs62098013	18	G	A	0.64	-0.01	2E-03	4.1E-09	-2.15E-02	2.23E-02	3.35E-01	/	/	/	/	/	/
rs62135536	2	C	T	0.97	0.04	6E-03	8E-10	-1.2E-01	5.22E-02	2.11E-02	-1.49E-03	2.06E-03	4.7E-01	-1.42E-02	2.48E-02	5.66E-01
rs62155874	2	A	G	0.87	-0.02	3E-03	5.2E-16	-4.13E-02	3.47E-02	2.35E-01	9.77E-04	1.09E-03	3.7E-01	-2.15E-02	1.24E-02	8.4E-02
rs62175972	2	T	C	0.97	0.03	6E-03	1.7E-08	-7.03E-02	8.04E-02	3.82E-01	2.77E-04	2E-03	8.9E-01	-6.2E-03	2.22E-02	7.8E-01
rs624833	4	T	G	0.70	0.01	2E-03	6.6E-10	-6.2E-03	2.22E-02	7.81E-01	7.71E-04	7.81E-04	3.2E-01	1.3E-02	8.7E-03	1.36E-01
rs6562474	13	C	G	0.65	0.01	2E-03	1E-08	4E-03	2.15E-02	8.53E-01	-1.2E-03	7.6E-04	1.1E-01	-9.2E-03	8.5E-03	2.79E-01
rs6598539	15	T	C	0.49	-0.01	2E-03	4.5E-09	1.5E-02	2.13E-02	4.83E-01	1.6E-03	7.22E-04	2.7E-02	2.52E-02	8.3E-03	2.43E-03
rs6741228	2	T	C	0.43	0.01	2E-03	1.6E-08	3.6E-03	2.15E-02	8.67E-01	1.42E-03	7.29E-04	5.2E-02	1.52E-02	8.3E-03	6.65E-02
rs67596067	17	G	A	0.65	-0.01	2E-03	1.2E-09	1.51E-02	2.36E-02	5.22E-01	6.73E-05	7.58E-04	9.3E-01	1.4E-02	8.6E-03	1.06E-01
rs6778080	3	T	C	0.27	0.02	2E-03	1.3E-12	-3.66E-02	2.58E-02	1.56E-01	-1.67E-03	8.16E-04	4.1E-02	-3.13E-02	8.9E-03	4.49E-04
rs6779302	3	G	T	0.63	-0.01	2E-03	1.2E-09	3.9E-03	2.22E-02	8.62E-01	5.57E-04	7.5E-04	4.6E-01	-8.7E-03	8.4E-03	3.01E-01
rs6935954	6	A	G	0.42	0.01	2E-03	8.2E-12	3.1E-03	2.25E-02	8.9E-01	-9.26E-04	7.29E-04	2E-01	-3.4E-03	8.1E-03	6.77E-01
rs6957896	7	C	T	0.50	-0.01	2E-03	4.5E-08	4.3E-03	2.17E-02	8.41E-01	3.92E-04	7.22E-04	5.9E-01	7.9E-03	8.6E-03	3.59E-01
rs6962772	7	A	G	0.85	0.02	3E-03	7.8E-09	-3.31E-02	2.53E-02	1.91E-01	-2.62E-03	9.97E-04	8.6E-03	-2.3E-03	1.08E-02	8.28E-01
rs7039819	9	G	A	0.43	0.01	2E-03	5.1E-10	5.3E-03	2.14E-02	8.05E-01	-1.45E-04	7.29E-04	8.4E-01	2.1E-03	8.6E-03	8.02E-01
rs7077678	10	C	T	0.62	0.01	2E-03	2.6E-09	5.85E-02	2.18E-02	7.25E-03	1.24E-03	7.47E-04	9.7E-02	4.98E-02	8.1E-03	8.01E-10
rs71367545	18	G	A	0.79	-0.02	2E-03	1.4E-09	8.1E-03	2.59E-02	7.54E-01	-2.25E-05	8.84E-04	9.8E-01	-3.7E-03	1.1E-02	7.37E-01
rs7155595	14	A	C	0.67	-0.01	2E-03	2.5E-09	2.48E-02	2.32E-02	2.85E-01	-3.67E-05	7.71E-04	9.6E-01	-1.25E-02	8.7E-03	1.51E-01

rs71627581	5	G	A	0.89	0.02	3E-03	1.6E-09	1.81E-02	2.84E-02	5.24E-01	2.19E-03	1.14E-03	5.6E-02	2.95E-02	1.31E-02	2.49E-02
rs72674867	8	A	T	0.77	0.01	2E-03	3.8E-08	-1.38E-02	2.21E-02	5.33E-01	7.85E-04	8.51E-04	3.6E-01	-9.7E-03	9.2E-03	2.9E-01
rs72678864	4	G	A	0.83	0.02	3E-03	1.6E-11	1.8E-02	3.07E-02	5.57E-01	1.42E-04	9.55E-04	8.8E-01	1.31E-02	1.14E-02	2.5E-01
rs7297175	12	T	C	0.43	-0.01	2E-03	6.6E-09	-1E-02	2.14E-02	6.41E-01	4.15E-04	7.28E-04	5.7E-01	-1.56E-02	8.2E-03	5.58E-02
rs732083	17	G	A	0.33	0.01	2E-03	1.5E-08	-4.59E-02	2.28E-02	4.44E-02	-2.08E-03	7.67E-04	6.8E-03	-1.89E-02	8.4E-03	2.43E-02
rs73220544	3	A	C	0.84	-0.02	3E-03	1.5E-08	-2.64E-02	2.47E-02	2.85E-01	2.14E-03	9.96E-04	3.1E-02	-1.3E-03	1.14E-02	9.1E-01
rs7333559	13	G	A	0.21	0.02	2E-03	3.2E-10	5.6E-03	2.88E-02	8.46E-01	1.36E-03	8.86E-04	1.3E-01	-1.59E-02	1.07E-02	1.35E-01
rs74086911	12	G	A	0.93	0.02	4E-03	2.1E-08	2.3E-03	4.64E-02	9.61E-01	2E-03	1.41E-03	1.5E-01	6.4E-03	1.56E-02	6.82E-01
rs7519626	1	C	T	0.32	0.01	2E-03	1.2E-08	1.1E-03	2.19E-02	9.61E-01	6.31E-04	7.71E-04	4.1E-01	-1.18E-02	8.6E-03	1.73E-01
rs7528604	1	G	A	0.57	0.01	2E-03	5.7E-12	-3.45E-02	2.2E-02	1.17E-01	1.32E-03	7.28E-04	7.00E-02	-3.3E-03	8.4E-03	6.98E-01
rs7553348	1	G	A	0.44	0.01	2E-03	5.2E-12	8.2E-03	2.12E-02	7E-01	4.1E-04	7.26E-04	5.7E-01	4.1E-03	8.1E-03	6.15E-01
rs7569203	2	A	C	0.69	-0.02	2E-03	7.4E-13	1.88E-02	2.18E-02	3.87E-01	8.13E-04	7.8E-04	3E-01	-5.2E-03	8.6E-03	5.46E-01
rs75742406	11	G	A	0.74	0.01	2E-03	1.3E-09	-3.24E-02	2.38E-02	1.73E-01	1.19E-03	8.23E-04	1.5E-01	9E-03	9.4E-03	3.41E-01
rs76608582	19	C	A	0.95	0.03	5E-03	3.2E-10	8.33E-02	4.56E-02	6.79E-02	4.84E-04	1.78E-03	7.9E-01	-4.22E-02	2.35E-02	7.26E-02
rs775758	3	A	T	0.43	0.01	2E-03	1.1E-08	5.22E-02	2.13E-02	1.45E-02	/	/	/	/	/	/
rs7766610	6	C	A	0.18	0.02	3E-03	2.2E-12	-3.28E-02	2.37E-02	1.66E-01	1.13E-03	9.34E-04	2.3E-01	1.7E-02	1.01E-02	9.18E-02
rs7807019	7	A	G	0.54	-0.02	2E-03	6.7E-14	-2.37E-02	2.15E-02	2.69E-01	3.86E-04	7.23E-04	5.9E-01	2E-03	8.2E-03	8.1E-01
rs8042134	15	T	G	0.54	-0.01	2E-03	1.3E-12	1.54E-02	2.14E-02	4.73E-01	-9.82E-05	7.28E-04	8.9E-01	2.1E-03	8.8E-03	8.13E-01
rs8042849	15	C	T	0.34	0.03	2E-03	1.8E-39	-2.37E-02	2.23E-02	2.88E-01	-5.83E-04	7.61E-04	4.4E-01	9.4E-03	8.4E-03	2.66E-01
rs860326	14	C	T	0.43	0.01	2E-03	2.7E-09	1.44E-02	2.15E-02	5.01E-01	7.61E-04	7.28E-04	3E-01	-2E-04	8.5E-03	9.84E-01
rs8614	17	C	A	0.82	-0.02	3E-03	1.8E-10	-5.19E-02	2.96E-02	8.01E-02	4.59E-04	9.34E-04	6.2E-01	-3.86E-02	1.11E-02	4.97E-04
rs889398	16	C	T	0.59	0.01	2E-03	6.3E-11	-6.4E-03	2.17E-02	7.67E-01	-1.97E-03	7.35E-04	7.4E-03	1E-03	8.2E-03	9.06E-01
rs9435340	1	T	A	0.34	0.01	2E-03	1.2E-08	2E-03	2.33E-02	9.33E-01	-2.51E-03	7.61E-04	9.6E-04	-3.5E-03	8.6E-03	6.84E-01
rs9842947	3	C	T	0.33	-0.01	2E-03	3.1E-09	2.06E-02	2.31E-02	3.73E-01	-2.99E-04	7.71E-04	7.00E-01	4.3E-03	8.7E-03	6.24E-01
rs986391	5	G	A	0.37	0.02	2E-03	9.4E-15	-4.1E-03	2.25E-02	8.55E-01	-2.61E-04	7.47E-04	7.3E-01	1.9E-03	9E-03	8.29E-01
rs9904288	17	T	C	0.71	0.01	2E-03	3.1E-08	6E-04	2.39E-02	9.79E-01	-1.06E-03	7.91E-04	1.8E-01	3.9E-03	8.8E-03	6.56E-01

	rs9919670	11	G	A	0.61	-0.02	2E-03	7.6E-27	1.2E-02	2.21E-02	5.86E-01	-1.7E-05	7.38E-04	9.8E-01	-6.5E-03	8.1E-03	4.27E-01
Light smoking	rs1864882	2	G	T	0.36	0.01	2E-03	1.8E-08	0.00E+00	2.28E-02	1E+00	-1.37E-04	7.52E-04	8.6E-01	2.2E-03	8.5E-03	7.98E-01
	rs1565735	8	A	T	0.21	-0.02	2E-03	1.8E-11	-2.75E-02	3E-02	3.6E-01	-1.3E-03	9.01E-04	1.5E-01	-1.13E-02	1.04E-02	2.78E-01
	rs2036527	15	A	G	0.32	-0.03	2E-03	1E-36	3.27E-02	2.26E-02	1.47E-01	5.5E-04	7.66E-04	4.7E-01	-1.04E-02	8.5E-03	2.23E-01
Smoking initiation	rs10001365	4	A	G	0.41	-0.02	3.64E-03	6.65E-12	5.1E-03	2.15E-02	8.12E-01	-4.78E-04	7.4E-04	5.2E-01	1.45E-02	8.1E-03	7.25E-02
	rs1004787	2	A	G	0.58	0.03	3.57E-03	5.27E-17	5E-02	2.19E-02	2.26E-02	8.52E-04	7.25E-04	2.4E-01	-1.08E-02	8.2E-03	1.87E-01
	rs10114490	9	A	G	0.20	-0.03	4.53E-03	1.81E-08	1.68E-02	2.45E-02	4.91E-01	-9.7E-05	9.2E-04	9.2E-01	4.8E-03	9.9E-03	6.26E-01
	rs10159545	10	G	C	0.38	0.03	3.73E-03	1.84E-12	-5.83E-02	2.22E-02	8.62E-03	-4.69E-04	7.6E-04	5.4E-01	-2.2E-03	8.3E-03	7.92E-01
	rs10233018	7	G	A	0.50	0.03	3.56E-03	2.75E-14	-2.62E-02	2.16E-02	2.26E-01	7.11E-04	7.21E-04	3.2E-01	-4.5E-03	8.2E-03	5.88E-01
	rs10260968	7	A	G	0.60	-0.02	3.61E-03	1.75E-08	1.66E-02	2.14E-02	4.39E-01	2.3E-03	7.32E-04	1.6E-03	2.51E-02	8.3E-03	2.62E-03
	rs10279261	7	A	G	0.62	-0.02	3.66E-03	5E-09	-1.19E-02	2.22E-02	5.94E-01	2.74E-04	7.44E-04	7.1E-01	-1.23E-02	8.3E-03	1.41E-01
	rs10498846	6	T	C	0.47	0.02	3.56E-03	6.62E-09	2.12E-02	2.14E-02	3.22E-01	4.95E-04	7.23E-04	4.9E-01	-3.1E-03	8.2E-03	7.08E-01
	rs1050847	16	T	C	0.51	-0.02	3.59E-03	1.67E-09	6E-03	2.14E-02	7.78E-01	-1.04E-03	7.3E-04	1.6E-01	3.3E-03	8.4E-03	6.92E-01
	rs10905461	10	C	T	0.72	-0.02	4.15E-03	7.35E-09	1.96E-02	2.89E-02	4.98E-01	-6.1E-04	8.23E-04	4.6E-01	1.67E-02	9.6E-03	8.06E-02
	rs11057005	12	G	A	0.43	-0.02	3.58E-03	4.85E-09	6.87E-02	2.16E-02	1.45E-03	3.7E-04	7.3E-04	6.1E-01	-2.5E-03	8.1E-03	7.56E-01
	rs11078713	17	G	A	0.45	-0.02	3.61E-03	2.23E-08	1.35E-02	2.15E-02	5.3E-01	1.72E-03	7.32E-04	1.9E-02	2.99E-02	8.7E-03	5.7E-04
	rs1154693	3	G	A	0.86	0.03	4.91E-03	3.12E-11	3E-03	2.6E-02	9.07E-01	7.07E-04	1.02E-03	4.9E-01	2.3E-02	1.11E-02	3.93E-02
	rs1160685	4	G	C	0.48	0.02	3.59E-03	7.2E-09	-1.74E-02	2.2E-02	4.3E-01	2.99E-04	7.23E-04	6.8E-01	-3.6E-03	8.1E-03	6.61E-01
	rs11658881	17	G	A	0.42	0.02	3.61E-03	2.43E-08	3.07E-02	2.18E-02	1.6E-01	-5.84E-04	7.3E-04	4.2E-01	7.00E-04	8.5E-03	9.39E-01
	rs11712680	3	C	A	0.17	-0.03	4.58E-03	3.51E-09	-1.56E-02	3.09E-02	6.15E-01	-4.14E-04	9.27E-04	6.5E-01	5.9E-03	1.08E-02	5.83E-01
	rs117143374	21	C	T	0.12	0.03	5.27E-03	2.76E-08	1.47E-02	3.46E-02	6.7E-01	-8.84E-04	1.04E-03	4E-01	2.05E-02	1.23E-02	9.6E-02
	rs11872397	18	A	G	0.25	-0.02	4.09E-03	1.43E-09	1.43E-02	2.57E-02	5.77E-01	-3.89E-04	8.32E-04	6.4E-01	1.65E-02	9.8E-03	9.24E-02
	rs12025237	1	C	A	0.12	-0.03	5.34E-03	6.52E-10	-1.7E-03	2.68E-02	9.51E-01	-8.43E-04	1.09E-03	4.4E-01	-2.9E-02	1.22E-02	1.76E-02
	rs12042107	1	C	T	0.53	-0.02	3.57E-03	4.22E-10	-4.59E-02	2.19E-02	3.61E-02	3.93E-04	7.25E-04	5.9E-01	7.6E-03	8.6E-03	3.77E-01
	rs12112638	7	G	A	0.28	-0.02	4.04E-03	1.34E-09	-2.52E-02	2.36E-02	2.85E-01	-1.98E-05	8.14E-04	9.8E-01	1.48E-02	9.2E-03	1.08E-01

rs12186738	5	T	G	0.15	-0.03	5.02E-03	3.42E-11	1.3E-02	3.02E-02	6.68E-01	2.01E-03	1.03E-03	5.1E-02	2.01E-02	1.14E-02	7.79E-02
rs12333760	7	C	T	0.20	-0.03	4.8E-03	1.44E-09	-3.41E-02	2.52E-02	1.75E-01	-2.25E-03	9.73E-04	2.1E-02	-1.6E-03	1.06E-02	8.8E-01
rs12356821	10	C	G	0.14	0.04	5.05E-03	6.27E-15	-4.61E-02	2.97E-02	1.21E-01	-2.27E-03	1.03E-03	2.8E-02	-4.02E-02	1.12E-02	3.25E-04
rs12441907	15	A	C	0.19	-0.03	4.52E-03	1.06E-10	-2.37E-02	2.74E-02	3.87E-01	-5.04E-04	9.24E-04	5.9E-01	-9E-03	1.02E-02	3.78E-01
rs12474587	2	T	G	0.40	0.03	3.58E-03	1.25E-14	-2.2E-03	2.23E-02	9.22E-01	3.63E-04	7.25E-04	6.2E-01	-8.9E-03	8.2E-03	2.74E-01
rs12545053	8	G	A	0.40	0.02	3.64E-03	2.43E-08	-4E-04	2.16E-02	9.87E-01	-4.57E-04	7.36E-04	5.3E-01	1.41E-02	8.7E-03	1.08E-01
rs12632110	3	G	A	0.65	-0.02	3.75E-03	4.78E-10	-7.8E-03	2.34E-02	7.38E-01	-7.00E-04	7.6E-04	3.6E-01	-2.17E-02	8.4E-03	1.02E-02
rs13030994	2	A	G	0.49	0.04	3.56E-03	3.56E-24	2.38E-02	2.15E-02	2.69E-01	9.7E-04	7.2E-04	1.8E-01	1E-04	8.1E-03	9.86E-01
rs13145728	4	C	G	0.36	-0.02	3.66E-03	2.14E-10	5.43E-02	2.21E-02	1.4E-02	1.29E-03	7.43E-04	8.3E-02	3.32E-02	8.4E-03	7.7E-05
rs13246563	7	G	C	0.53	-0.02	3.74E-03	3.45E-10	2.89E-02	2.19E-02	1.86E-01	9.05E-04	7.26E-04	2.1E-01	-1.37E-02	8E-03	8.69E-02
rs13261666	8	T	G	0.52	-0.03	3.56E-03	3.9E-14	2.91E-02	2.14E-02	1.74E-01	-4.06E-06	7.21E-04	1E+00	4.8E-03	8.5E-03	5.75E-01
rs134529	22	C	T	0.35	-0.02	3.66E-03	4.85E-08	1.53E-02	2.27E-02	5.01E-01	3.07E-04	7.41E-04	6.8E-01	-4.6E-03	8.3E-03	5.81E-01
rs1385108	5	T	C	0.24	0.02	4.16E-03	3E-09	-7.9E-03	2.37E-02	7.39E-01	-2.4E-04	8.44E-04	7.8E-01	-7.5E-03	9.4E-03	4.21E-01
rs1435741	15	A	G	0.43	0.03	3.59E-03	2.64E-16	-4.63E-02	2.19E-02	3.46E-02	7.11E-04	7.28E-04	3.3E-01	-1.09E-02	8E-03	1.73E-01
rs1445649	2	C	T	0.53	0.02	3.56E-03	1.68E-11	-2.9E-03	2.14E-02	8.91E-01	-3.71E-04	7.23E-04	6.1E-01	-7.7E-03	8.1E-03	3.37E-01
rs1555445	20	T	A	0.34	0.02	3.82E-03	3.65E-09	9E-04	2.16E-02	9.66E-01	5.84E-04	7.84E-04	4.6E-01	-8.5E-03	8.7E-03	3.3E-01
rs1565735	8	A	T	0.21	-0.04	4.46E-03	3.42E-17	-2.75E-02	3E-02	3.6E-01	-1.3E-03	9.01E-04	1.5E-01	-1.13E-02	1.04E-02	2.78E-01
rs1869243	3	C	T	0.48	0.02	3.56E-03	2.97E-08	9.3E-03	2.13E-02	6.62E-01	-1.64E-04	7.23E-04	8.2E-01	1.07E-02	8.1E-03	1.84E-01
rs1899896	8	T	C	0.29	0.03	3.89E-03	1.04E-11	3.01E-02	2.42E-02	2.14E-01	4.68E-04	7.92E-04	5.6E-01	9.5E-03	8.8E-03	2.77E-01
rs1971318	12	T	C	0.14	0.03	4.93E-03	7.06E-09	-3.69E-02	3.32E-02	2.67E-01	7.04E-04	9.91E-04	4.8E-01	-2.78E-02	1.14E-02	1.43E-02
rs2046850	1	T	C	0.19	-0.02	4.48E-03	3.03E-08	3.11E-02	2.38E-02	1.91E-01	9.14E-04	9.14E-04	3.2E-01	7.9E-03	1E-02	4.33E-01
rs2050586	1	C	G	0.36	-0.02	3.71E-03	3E-08	7.14E-02	2.3E-02	1.85E-03	-1.33E-03	7.54E-04	7.7E-02	-8E-04	8.6E-03	9.28E-01
rs2107300	2	G	C	0.85	-0.03	4.93E-03	3.27E-08	-3.4E-03	2.99E-02	9.1E-01	-1.36E-03	9.95E-04	1.7E-01	-5.3E-03	1.12E-02	6.35E-01
rs2186122	1	T	A	0.56	0.03	3.59E-03	3.61E-13	2.31E-02	2.18E-02	2.89E-01	-1.22E-03	7.29E-04	9.4E-02	1.6E-03	8.4E-03	8.45E-01
rs222449	6	T	A	0.79	-0.03	4.43E-03	1.08E-08	1.75E-02	2.56E-02	4.95E-01	1.9E-03	9.07E-04	3.6E-02	-1.11E-02	1.03E-02	2.83E-01

rs2378662	9	A	G	0.56	0.02	3.57E-03	4.16E-09	-2.32E-02	2.16E-02	2.82E-01	-4.31E-04	7.27E-04	5.5E-01	-7.00E-03	8.3E-03	3.96E-01
rs240963	6	C	T	0.84	-0.04	4.84E-03	2.16E-17	-3.67E-02	2.69E-02	1.72E-01	8.69E-04	9.89E-04	3.8E-01	1.93E-02	1.09E-02	7.75E-02
rs2631024	8	G	A	0.74	-0.02	4.03E-03	1.18E-08	-6E-03	2.3E-02	7.94E-01	2.6E-04	8.26E-04	7.5E-01	-1.3E-02	9E-03	1.51E-01
rs266047	2	A	G	0.53	-0.03	3.74E-03	3.36E-16	-8E-04	2.13E-02	9.7E-01	1.9E-04	7.22E-04	7.9E-01	-7.00E-03	8E-03	3.83E-01
rs2678897	2	A	G	0.63	0.02	3.64E-03	3.51E-08	1.32E-02	2.2E-02	5.5E-01	-6.9E-05	7.4E-04	9.3E-01	1.55E-02	8.2E-03	5.77E-02
rs3001723	1	A	G	0.32	0.03	3.9E-03	8.12E-18	-1.32E-02	2.41E-02	5.85E-01	-7.69E-04	7.86E-04	3.3E-01	-2.49E-02	8.9E-03	5.14E-03
rs301805	1	G	T	0.56	0.02	3.61E-03	2.8E-09	1.82E-02	2.23E-02	4.16E-01	1.02E-03	7.3E-04	1.6E-01	3.4E-03	8.5E-03	6.85E-01
rs35702515	2	T	G	0.16	0.03	4.23E-03	2.43E-09	2.36E-02	2.84E-02	4.06E-01	-3.83E-04	8.64E-04	6.6E-01	-1.31E-02	1.01E-02	1.95E-01
rs3800227	6	G	A	0.70	0.02	4.06E-03	1.93E-08	5.6E-03	2.28E-02	8.04E-01	4.7E-04	8.26E-04	5.7E-01	1.37E-02	9.3E-03	1.41E-01
rs3801289	7	C	A	0.35	-0.02	3.74E-03	3.74E-09	-1.5E-02	2.15E-02	4.85E-01	-5.37E-04	7.64E-04	4.8E-01	-4.3E-03	9.5E-03	6.52E-01
rs3904512	13	A	G	0.43	-0.02	3.58E-03	3.23E-09	-4.25E-02	2.17E-02	5.01E-02	4.19E-04	7.24E-04	5.6E-01	5E-03	8.2E-03	5.39E-01
rs4044321	5	G	A	0.64	-0.03	3.71E-03	6.08E-14	-1.1E-02	2.26E-02	6.25E-01	-2.22E-04	7.52E-04	7.7E-01	3.6E-03	9E-03	6.9E-01
rs4236259	7	G	T	0.50	-0.02	3.56E-03	3.35E-12	1.3E-03	2.15E-02	9.53E-01	-5.95E-04	7.29E-04	4.1E-01	-3E-03	8.7E-03	7.28E-01
rs4352629	5	T	C	0.49	-0.03	3.57E-03	1.22E-14	-2.05E-02	2.14E-02	3.39E-01	1.74E-03	7.23E-04	1.6E-02	1.32E-02	7.9E-03	9.67E-02
rs4523689	11	G	A	0.41	-0.02	3.64E-03	1.55E-08	-4E-03	2.13E-02	8.52E-01	-2.8E-06	7.38E-04	1E+00	-3.4E-03	8.4E-03	6.84E-01
rs4543592	9	C	T	0.47	0.02	3.56E-03	7.46E-10	-3E-04	2.18E-02	9.89E-01	-6.16E-04	7.22E-04	3.9E-01	-4.4E-03	8.1E-03	5.86E-01
rs4674993	2	G	A	0.21	-0.03	4.44E-03	1.32E-08	5.38E-02	2.78E-02	5.34E-02	9.83E-04	9.02E-04	2.8E-01	-1.18E-02	1.05E-02	2.6E-01
rs4759228	12	C	G	0.27	-0.02	3.93E-03	3.58E-08	-2.08E-02	2.49E-02	4.03E-01	-3.12E-04	7.9E-04	6.9E-01	8E-03	9.5E-03	4.03E-01
rs4781977	16	C	T	0.21	-0.02	4.36E-03	4.54E-08	2.13E-02	3.66E-02	5.6E-01	1.32E-03	8.74E-04	1.3E-01	3.3E-02	1.03E-02	1.41E-03
rs4785836	16	C	T	0.40	-0.02	3.66E-03	2.26E-08	6E-03	2.16E-02	7.8E-01	-1.66E-04	7.46E-04	8.2E-01	-2E-04	8.2E-03	9.84E-01
rs6265	11	T	C	0.20	-0.03	4.58E-03	3.77E-12	4.47E-02	2.94E-02	1.29E-01	2.05E-03	9.21E-04	2.6E-02	5E-04	1.02E-02	9.64E-01
rs6433897	2	C	T	0.75	0.02	4.06E-03	3.16E-08	4.1E-03	2.44E-02	8.66E-01	1.53E-03	8.2E-04	6.2E-02	1.71E-02	9.2E-03	6.34E-02
rs6508144	18	G	C	0.56	-0.02	3.59E-03	7.97E-09	-4.9E-03	2.15E-02	8.21E-01	-2.06E-04	7.28E-04	7.8E-01	8.6E-03	8.6E-03	3.2E-01
rs66680800	3	T	G	0.40	-0.02	3.65E-03	2.83E-08	-2.67E-02	2.17E-02	2.19E-01	1.19E-04	7.34E-04	8.7E-01	-5.9E-03	8.3E-03	4.77E-01
rs6669839	1	T	C	0.20	0.03	4.4E-03	3.36E-09	-2.18E-02	2.69E-02	4.16E-01	-4.53E-04	8.88E-04	6.1E-01	-1.3E-03	1.03E-02	9E-01

	rs6728726	2	C	T	0.83	0.04	4.73E-03	6.73E-14	3.28E-02	2.86E-02	2.53E-01	-1.68E-03	9.51E-04	7.7E-02	-3.05E-02	1.05E-02	3.79E-03
	rs6788098	3	T	A	0.62	-0.03	3.69E-03	1.91E-17	4E-02	2.37E-02	9.17E-02	3.62E-04	7.44E-04	6.3E-01	-1.44E-02	8.4E-03	8.47E-02
	rs6893752	5	G	A	0.77	-0.02	4.07E-03	3.25E-09	-8E-04	2.48E-02	9.75E-01	-8.75E-04	8.24E-04	2.9E-01	-7.00E-04	9.2E-03	9.42E-01
	rs7197072	16	T	C	0.24	-0.02	4.17E-03	2.77E-09	-2.22E-02	2.22E-02	3.17E-01	1.22E-03	8.63E-04	1.6E-01	1.32E-02	9.7E-03	1.71E-01
	rs7224742	17	T	C	0.60	-0.02	3.66E-03	1.43E-08	1.74E-02	2.24E-02	4.37E-01	-4.5E-04	7.42E-04	5.4E-01	-1.7E-03	8.2E-03	8.37E-01
	rs72789632	5	T	C	0.12	-0.03	5.29E-03	5.02E-10	1.09E-02	3.82E-02	7.75E-01	2.21E-03	1.08E-03	4E-02	-6.8E-03	1.22E-02	5.77E-01
	rs72896886	18	C	G	0.14	-0.03	4.84E-03	2.75E-08	1.3E-02	2.87E-02	6.5E-01	-7.35E-04	9.7E-04	4.5E-01	1.26E-02	1.13E-02	2.67E-01
	rs7322872	13	T	C	0.78	-0.03	4.33E-03	3.58E-09	3.8E-03	2.81E-02	8.93E-01	1.6E-03	8.84E-04	7.1E-02	-1.45E-02	1.07E-02	1.76E-01
	rs7555507	1	T	C	0.50	-0.02	3.56E-03	1.14E-11	-1.94E-02	2.13E-02	3.63E-01	7.49E-05	7.21E-04	9.2E-01	-7.5E-03	8E-03	3.46E-01
	rs7585579	2	G	C	0.51	0.02	3.73E-03	1.88E-09	-1.46E-02	2.14E-02	4.96E-01	-2.42E-04	7.28E-04	7.4E-01	1.5E-02	8.1E-03	6.44E-02
	rs76214862	14	C	A	0.20	-0.02	4.55E-03	3.99E-08	-2.49E-02	2.54E-02	3.27E-01	1.47E-03	9.26E-04	1.1E-01	3.1E-03	1.02E-02	7.6E-01
	rs76608582	19	A	C	0.04	-0.05	8.26E-03	1.94E-09	8.33E-02	4.56E-02	6.79E-02	4.84E-04	1.78E-03	7.9E-01	-4.22E-02	2.35E-02	7.26E-02
	rs7921378	10	C	G	0.46	-0.03	3.56E-03	8.26E-13	-2.14E-02	2.13E-02	3.15E-01	-7.49E-04	7.24E-04	3E-01	-1.37E-02	8.4E-03	1.05E-01
	rs7929518	11	G	A	0.77	0.02	4.28E-03	1.56E-08	-2.15E-02	2.45E-02	3.79E-01	-1.18E-03	8.69E-04	1.8E-01	-1.23E-02	9.4E-03	1.88E-01
	rs7938812	11	G	T	0.42	0.04	3.64E-03	2.71E-33	1.16E-02	2.21E-02	5.98E-01	-2.18E-05	7.39E-04	9.8E-01	-6.9E-03	8.1E-03	3.96E-01
	rs7969559	12	G	A	0.69	-0.02	3.96E-03	7.31E-10	-1.12E-02	2.23E-02	6.16E-01	-6.6E-04	8E-04	4.1E-01	-2.3E-03	9.4E-03	8.1E-01
	rs9401770	6	A	G	0.27	0.03	3.99E-03	3.47E-12	-1.53E-02	2.26E-02	4.98E-01	9.39E-05	8.15E-04	9.1E-01	1.2E-03	9.3E-03	8.94E-01
	rs9423279	10	G	C	0.64	-0.02	3.71E-03	3.21E-08	2.43E-02	2.2E-02	2.69E-01	9.09E-04	7.73E-04	2.4E-01	-2.5E-03	1E-02	8.05E-01
	rs9540729	13	T	A	0.50	-0.02	3.56E-03	3.82E-08	1.79E-02	2.15E-02	4.06E-01	-8.3E-04	7.21E-04	2.5E-01	3.7E-03	8.2E-03	6.48E-01
	rs962625	4	G	A	0.24	0.02	4.04E-03	4.37E-09	-2.76E-02	2.66E-02	3E-01	-1.59E-03	8.19E-04	5.2E-02	-5.8E-03	9.3E-03	5.33E-01
	rs993700	4	C	T	0.77	-0.03	4.29E-03	1.53E-09	4.2E-03	2.52E-02	8.69E-01	6.25E-04	8.68E-04	4.7E-01	-2.5E-03	9.6E-03	7.92E-01
Amount of smoking per day	rs11725618	4	C	T	0.29	0.04	6.16E-03	4.67E-09	-2.49E-02	2.4E-02	2.99E-01	7.56E-04	8.32E-04	3.6E-01	2.1E-03	9.7E-03	8.29E-01
	rs11852372	15	C	A	0.33	0.18	5.91E-03	1E-200	3.12E-02	2.25E-02	1.66E-01	4.86E-04	7.67E-04	5.3E-01	-8.9E-03	8.6E-03	3.01E-01
	rs1579233	16	G	A	0.57	-0.03	5.56E-03	1.07E-08	1.13E-02	2.26E-02	6.16E-01	8.82E-04	7.26E-04	2.2E-01	1.39E-02	8.1E-03	8.58E-02
	rs2072659	1	G	C	0.11	-0.07	9.25E-03	1.71E-12	7.45E-02	3.56E-02	3.61E-02	1.86E-03	1.24E-03	1.3E-01	2.89E-02	1.4E-02	3.85E-02

rs2084533	3	T	C	0.32	0.03	5.9E-03	1.22E-08	-1.58E-02	2.32E-02	4.95E-01	-2.47E-04	7.83E-04	7.5E-01	-6.6E-03	8.8E-03	4.53E-01
rs215600	7	A	G	0.64	-0.05	5.75E-03	1.1E-17	-1.1E-03	2.37E-02	9.64E-01	1.25E-03	7.53E-04	9.7E-02	-1.31E-02	8.6E-03	1.27E-01
rs2273500	20	C	T	0.16	0.07	7.8E-03	2.47E-18	-2.19E-02	2.62E-02	4.04E-01	-3.76E-04	1.02E-03	7.1E-01	-2.31E-02	1.19E-02	5.2E-02
rs2424888	20	A	G	0.41	0.03	5.64E-03	2.76E-09	-8.6E-03	2.13E-02	6.86E-01	4.96E-05	7.56E-04	9.5E-01	-6.4E-03	8.3E-03	4.43E-01
rs3025383	9	C	T	0.18	-0.06	7.05E-03	2.22E-16	6.25E-02	2.81E-02	2.61E-02	5.1E-04	9.38E-04	5.9E-01	4.9E-03	1.06E-02	6.45E-01
rs34406232	19	A	C	0.03	-0.15	1.67E-02	1.33E-18	1.94E-02	6.77E-02	7.74E-01	4.72E-04	2.25E-03	8.3E-01	9E-03	2.76E-02	7.44E-01
rs4785587	16	A	G	0.51	-0.03	5.53E-03	1.27E-09	-1.7E-03	2.22E-02	9.38E-01	-1.12E-03	7.3E-04	1.3E-01	-1E-03	8.3E-03	9.05E-01
rs56113850	19	C	T	0.57	0.11	5.6E-03	1.1E-81	2.2E-02	2.13E-02	3.02E-01	-8.4E-04	7.29E-04	2.5E-01	-1.04E-02	9.1E-03	2.53E-01
rs58379124	8	C	T	0.75	0.07	6.5E-03	9E-25	-4.5E-03	2.5E-02	8.58E-01	-1.8E-03	8.55E-04	3.5E-02	-5.1E-03	9.6E-03	5.98E-01
rs632811	15	G	A	0.35	-0.04	6.41E-03	1.03E-08	1.36E-02	2.13E-02	5.24E-01	4E-04	7.69E-04	6E-01	3.2E-03	8.7E-03	7.1E-01
rs73229090	8	A	C	0.11	0.06	8.76E-03	2.44E-10	-7.2E-03	3.64E-02	8.44E-01	-1.08E-03	1.13E-03	3.4E-01	-1.66E-02	1.33E-02	2.12E-01
rs7431710	3	A	G	0.64	-0.03	5.81E-03	1.82E-09	6.9E-03	2.25E-02	7.59E-01	-1.45E-03	7.58E-04	5.6E-02	-1.72E-02	8.3E-03	3.74E-02
rs75494138	11	T	C	0.06	0.06	1.06E-02	1.45E-08	-7.35E-02	5.32E-02	1.67E-01	-3.47E-03	1.39E-03	1.3E-02	-5.16E-02	1.67E-02	2.02E-03
rs787362	4	A	T	0.45	0.03	5.57E-03	4.5E-08	-2.13E-02	2.13E-02	3.18E-01	-6.18E-04	7.34E-04	4E-01	3.6E-03	8.1E-03	6.55E-01
rs790564	8	C	A	0.72	-0.04	6.19E-03	3.97E-11	-2.11E-02	2.49E-02	3.96E-01	-1.6E-03	8.09E-04	4.9E-02	2.3E-03	9.5E-03	8.07E-01
rs7928017	11	A	C	0.41	-0.03	5.56E-03	3.14E-09	-2.03E-02	2.36E-02	3.9E-01	-1.73E-04	7.27E-04	8.1E-01	-1.16E-02	8.4E-03	1.68E-01
rs7951365	11	C	T	0.31	0.04	5.97E-03	6.63E-11	1.91E-02	2.48E-02	4.42E-01	-9.1E-04	7.84E-04	2.5E-01	7.00E-04	8.7E-03	9.37E-01
rs806798	6	C	T	0.54	-0.03	5.53E-03	2.48E-08	9.9E-03	2.18E-02	6.49E-01	9.23E-04	7.21E-04	2E-01	1.19E-02	8E-03	1.38E-01
rs895330	19	G	C	0.21	-0.04	7.02E-03	2.68E-08	-1.7E-03	2.87E-02	9.54E-01	-1.71E-03	9.14E-04	6.2E-02	7.00E-04	1.07E-02	9.46E-01

SNP, single-nucleotide polymorphism; EA, effect allele; OA, other allele; EAF, effect allele frequency; SE, standard error;PCa, prostate cancer; PRACTICA, Prostate Cancer Association Group to Investigate Cancer-Associated Alterations in the Genome.

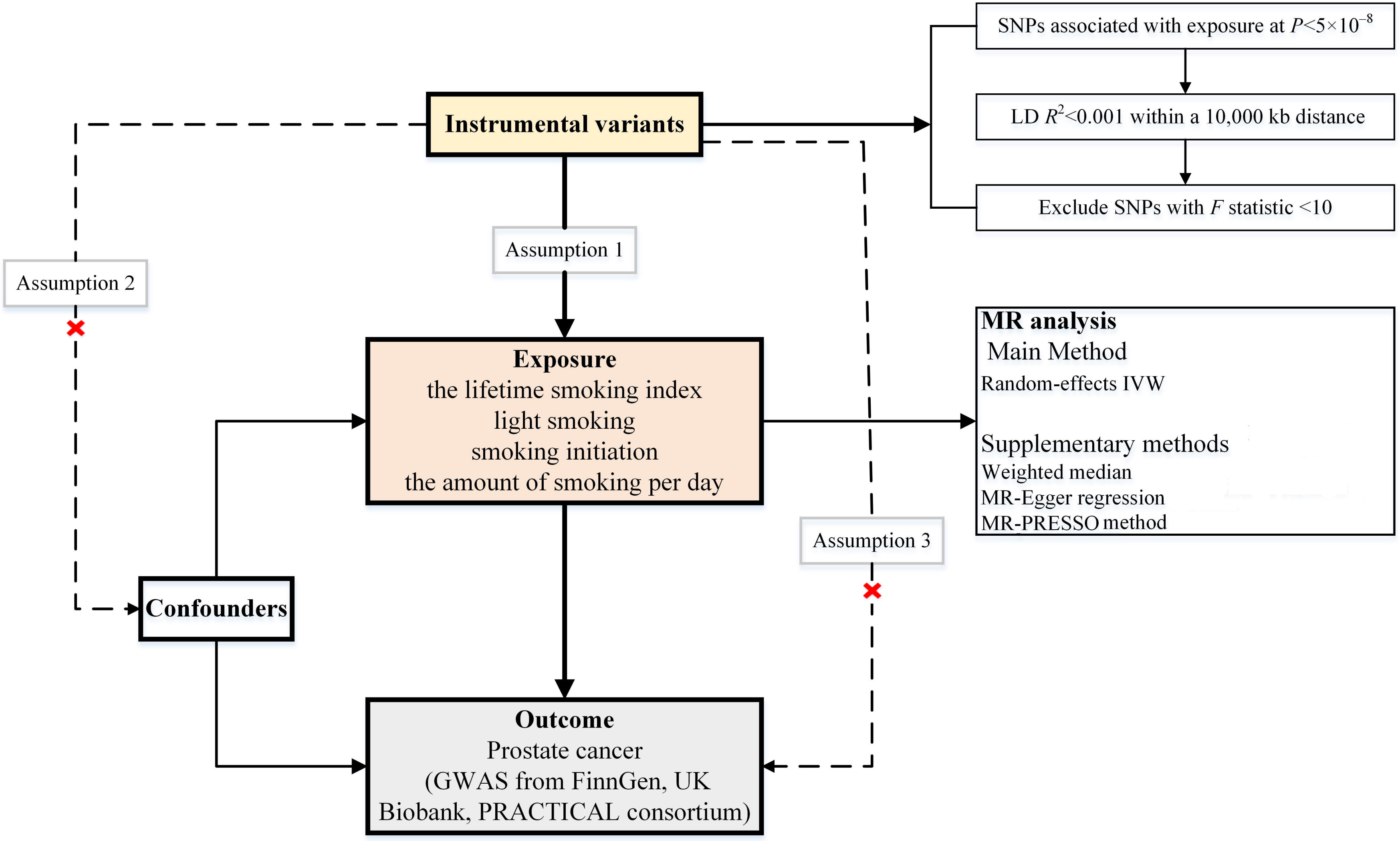

Instrumental variants
Assumption 1
Assumption 2
Assumption 3
Exposure
the lifetime smoking index
light smoking
smoking initiation
the amount of smoking per day
Confounders
Outcome
Prostate cancer
(GWAS from FinnGen, UK Biobank, PRACTICAL consortium)
SNPs associated with exposure at $P<5\times10^{-8}$
LD R^2<0.001 within a 10,000 kb distance
Exclude SNPs with F statistic <10
MR analysis
Main Method
Random-effects IVW
Supplementary methods
Weighted median
MR-Egger regression
MR-PRESSO method

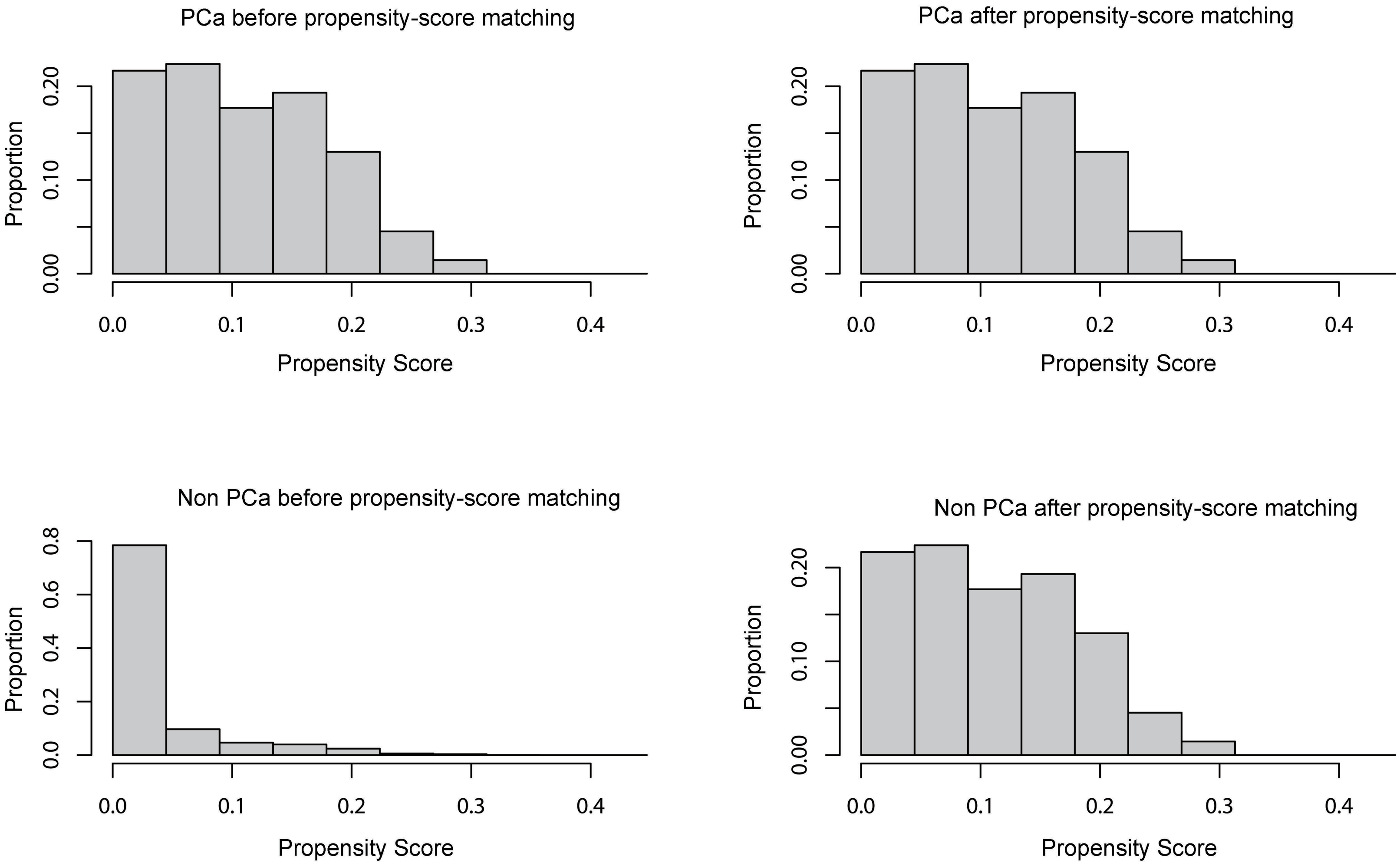
PCa before propensity-score matching
PCa after propensity-score matching
Non PCa before propensity-score matching
Non PCa after propensity-score matching
Proportion
Propensity Score
0.00
0.10
0.20
0.0
0.2
0.4
0.6
0.8
0.1
0.3

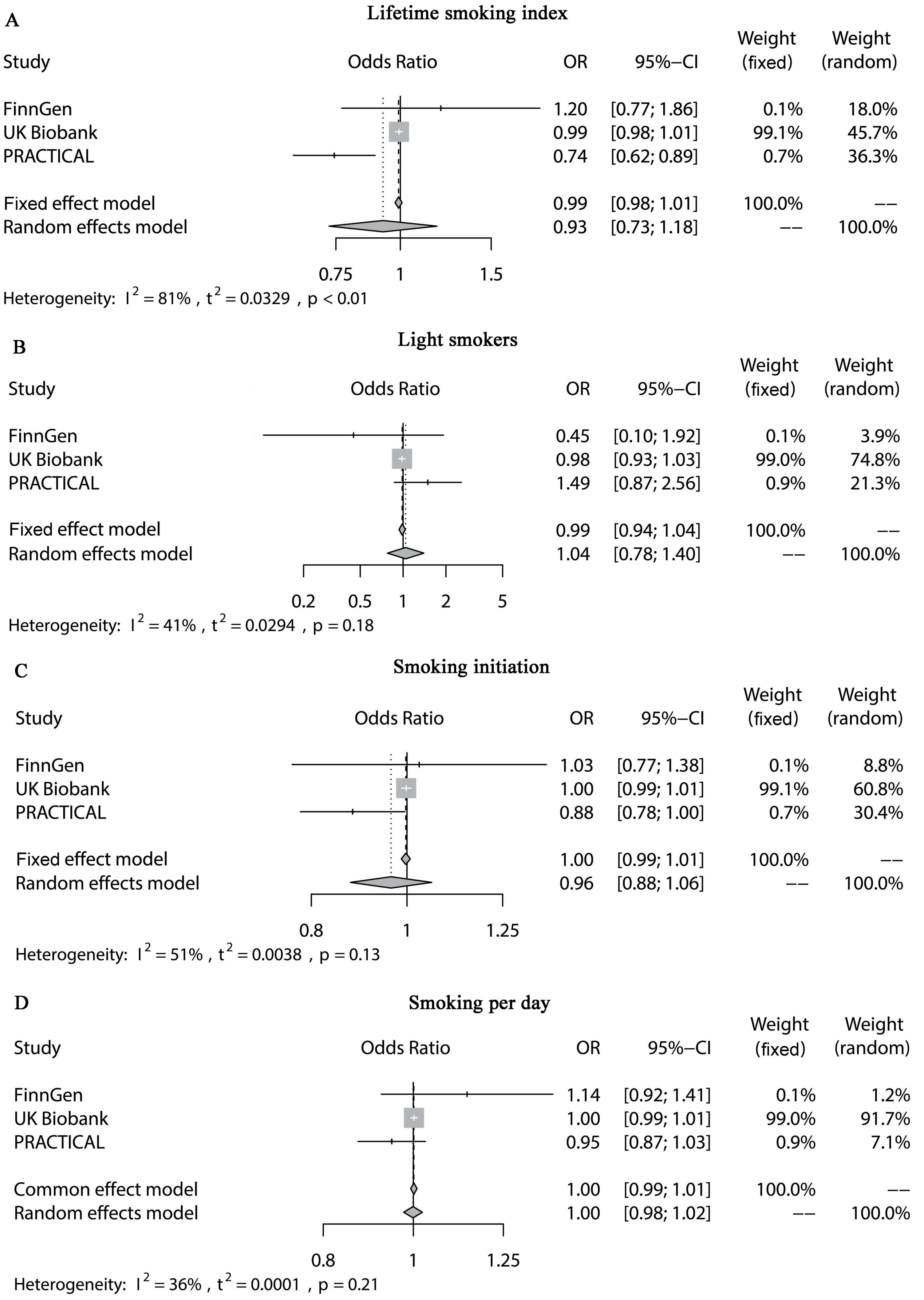
A
Lifetime smoking index
Study | Odds Ratio | OR | 95%−CI | Weight (fixed) | Weight (random)
FinnGen 1.20 [0.77; 1.86] 0.1% 18.0%
UK Biobank 0.99 [0.98; 1.01] 99.1% 45.7%
PRACTICAL 0.74 [0.62; 0.89] 0.7% 36.3%
Fixed effect model 0.99 [0.98; 1.01] 100.0% --
Random effects model 0.93 [0.73; 1.18] -- 100.0%
0.75 1 1.5
Heterogeneity: $I^2 = 81\%$, $t^2 = 0.0329$, $p < 0.01$
B
Light smokers
Study | Odds Ratio | OR | 95%−CI | Weight (fixed) | Weight (random)
FinnGen 0.45 [0.10; 1.92] 0.1% 3.9%
UK Biobank 0.98 [0.93; 1.03] 99.0% 74.8%
PRACTICAL 1.49 [0.87; 2.56] 0.9% 21.3%
Fixed effect model 0.99 [0.94; 1.04] 100.0% --
Random effects model 1.04 [0.78; 1.40] -- 100.0%
0.2 0.5 1 2 5
Heterogeneity: $I^2 = 41\%$, $t^2 = 0.0294$, $p = 0.18$
C
Smoking initiation
Study | Odds Ratio | OR | 95%−CI | Weight (fixed) | Weight (random)
FinnGen 1.03 [0.77; 1.38] 0.1% 8.8%
UK Biobank 1.00 [0.99; 1.01] 99.1% 60.8%
PRACTICAL 0.88 [0.78; 1.00] 0.7% 30.4%
Fixed effect model 1.00 [0.99; 1.01] 100.0% --
Random effects model 0.96 [0.88; 1.06] -- 100.0%
0.8 1 1.25
Heterogeneity: $I^2 = 51\%$, $t^2 = 0.0038$, $p = 0.13$
D
Smoking per day
Study | Odds Ratio | OR | 95%−CI | Weight (fixed) | Weight (random)
FinnGen 1.14 [0.92; 1.41] 0.1% 1.2%
UK Biobank 1.00 [0.99; 1.01] 99.0% 91.7%
PRACTICAL 0.95 [0.87; 1.03] 0.9% 7.1%
Common effect model 1.00 [0.99; 1.01] 100.0% --
Random effects model 1.00 [0.98; 1.02] -- 100.0%
0.8 1 1.25
Heterogeneity: $I^2 = 36\%$, $t^2 = 0.0001$, $p = 0.21$

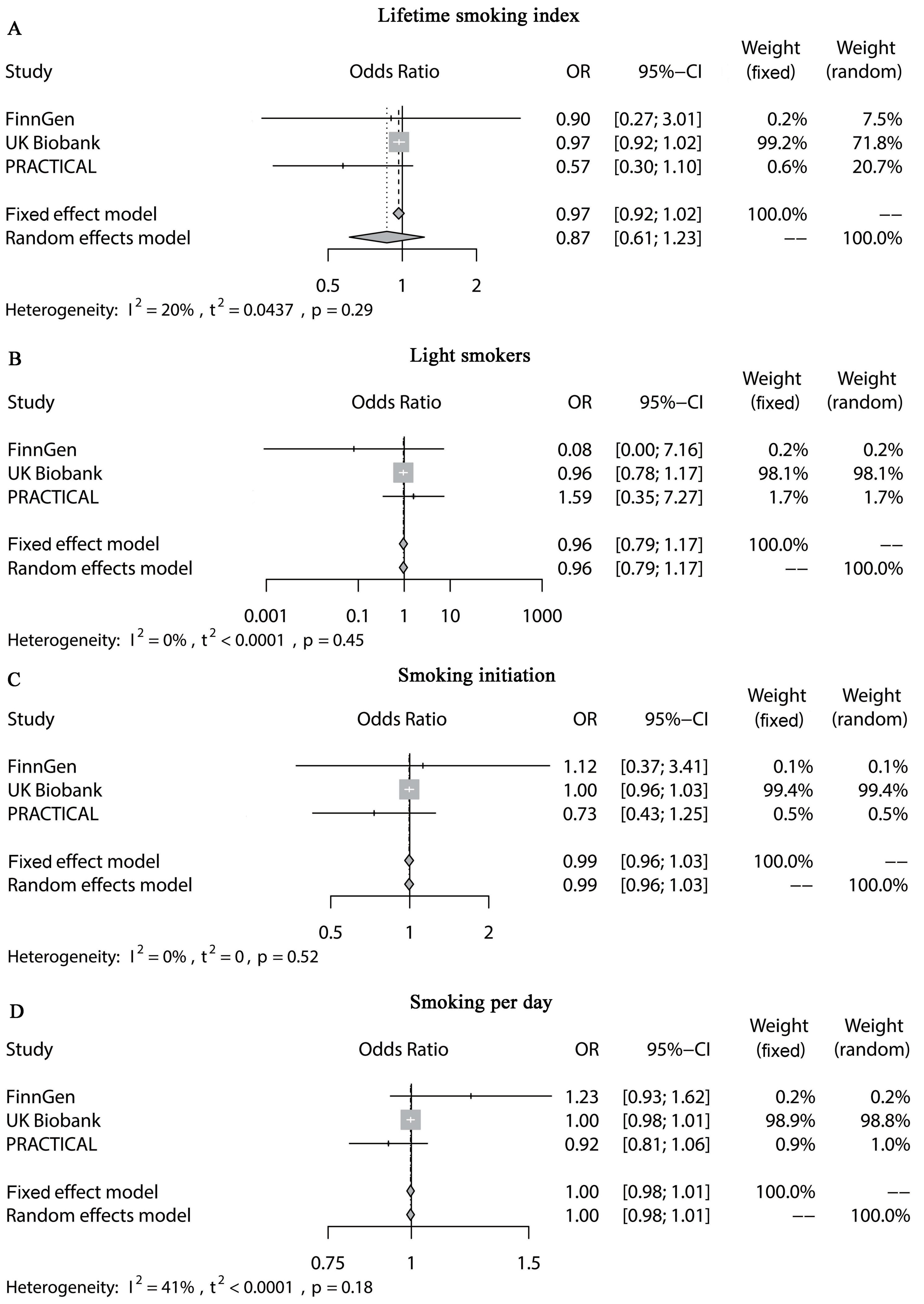
A
Lifetime smoking index
Study
Odds Ratio
OR
95%−CI
Weight (fixed)
Weight (random)
FinnGen 0.90 [0.27; 3.01] 0.2% 7.5%
UK Biobank 0.97 [0.92; 1.02] 99.2% 71.8%
PRACTICAL 0.57 [0.30; 1.10] 0.6% 20.7%
Fixed effect model 0.97 [0.92; 1.02] 100.0% --
Random effects model 0.87 [0.61; 1.23] -- 100.0%
0.5
1
2
Heterogeneity: $I^2 = 20\%$, $t^2 = 0.0437$, $p = 0.29$
B
Light smokers
Study
Odds Ratio
OR
95%−CI
Weight (fixed)
Weight (random)
FinnGen 0.08 [0.00; 7.16] 0.2% 0.2%
UK Biobank 0.96 [0.78; 1.17] 98.1% 98.1%
PRACTICAL 1.59 [0.35; 7.27] 1.7% 1.7%
Fixed effect model 0.96 [0.79; 1.17] 100.0% --
Random effects model 0.96 [0.79; 1.17] -- 100.0%
0.001
0.1
1
10
1000
Heterogeneity: $I^2 = 0\%$, $t^2 < 0.0001$, $p = 0.45$
C
Smoking initiation
Study
Odds Ratio
OR
95%−CI
Weight (fixed)
Weight (random)
FinnGen 1.12 [0.37; 3.41] 0.1% 0.1%
UK Biobank 1.00 [0.96; 1.03] 99.4% 99.4%
PRACTICAL 0.73 [0.43; 1.25] 0.5% 0.5%
Fixed effect model 0.99 [0.96; 1.03] 100.0% --
Random effects model 0.99 [0.96; 1.03] -- 100.0%
0.5
1
2
Heterogeneity: $I^2 = 0\%$, $t^2 = 0$, $p = 0.52$
D
Smoking per day
Study
Odds Ratio
OR
95%−CI
Weight (fixed)
Weight (random)
FinnGen 1.23 [0.93; 1.62] 0.2% 0.2%
UK Biobank 1.00 [0.98; 1.01] 98.9% 98.8%
PRACTICAL 0.92 [0.81; 1.06] 0.9% 1.0%
Fixed effect model 1.00 [0.98; 1.01] 100.0% --
Random effects model 1.00 [0.98; 1.01] -- 100.0%
0.75
1
1.5
Heterogeneity: $I^2 = 41\%$, $t^2 < 0.0001$, $p = 0.18$

Supplementary Table 2. The results of pleiotropy test and MR-PRESSO.

**Exposure**	**n.SNP**	**F-value**	***P* for global test**	**MR-PRESSO**			**MR-PRESSO (Outlier-corrected)**			***P* for distortion test**
				P	beta	SE	*P*	beta	SE	
**finn-b-C3_PROSTATE_EXALLC**										
Lifetime smoking index	126	21.78-196	0.517	0.7406	-0.0478	0.1439	/	/	/	/
Smoking initiation	92	29.81-144.74	0.084	0.3915	-0.0914	0.1062	/	/	/	/
Smoking per day	23	29.9-953.27	0.443	0.9645	-0.0035	0.0782	/	/	/	/
**ieu-b-4809 (UKB)**										
Lifetime smoking index	122	21.78-196	<0.001	0.403	0.0054	0.0065	0.292	0.0067	0.0063	0.879
Smoking initiation	92	29.81-144.74	0.039	0.1444	-0.0054	0.0037	/	/	/	/
Smoking per day	23	29.9-953.27	0.044	0.3612	-0.0033	0.0035	/	/	/	/
**ieu-b-85 (PRACTICAL)**										
Lifetime smoking index	122	21.78-196	<0.001	0.0326	0.1763	0.0816	0.0127	0.1856	0.0733	0.897
Smoking initiation	92	29.81-144.74	<0.001	0.9449	0.0035	0.0508	0.4249	0.0377	0.047	0.692
Smoking per day	23	29.9-953.27	0.133	0.0623	-0.0739	0.0376	/	/	/	/

SNP, single-nucleotide polymorphism; MR, Mendelian randomization; PRESSO,pleiotropy residual sum and outlier; SE, standard error; UKB,UK Biobank; PRACTICAL, Prostate Cancer Association Group to Investigate Cancer-Associated Alterations in the Genome

© 2024 He H. et al.
